# Is There a Classical Nonsense-Mediated Decay Pathway in Trypanosomes?

**DOI:** 10.1371/journal.pone.0025112

**Published:** 2011-09-21

**Authors:** Praveen Delhi, Rafael Queiroz, Diana Inchaustegui, Mark Carrington, Christine Clayton

**Affiliations:** 1 Zentrum für Molekulare Biologie der Universität Heidelberg, DKFZ-ZMBH Alliance, Hedielberg, Germany; 2 Department of Biochemistry, University of Cambridge, Cambridge, United Kingdom; The University of Maryland, United States of America

## Abstract

In many eukaryotes, messenger RNAs with premature termination codons are destroyed by a process called “nonsense-mediated decay”, which requires the RNA helicase Upf1 and also, usually, an interacting factor, Upf2. Recognition of premature termination codons may rely on their distance from either a splice site or the polyadenylation site, and long 3′-untranslated regions can trigger mRNA decay. The protist *Trypanosoma brucei* relies heavily on mRNA degradation to determine mRNA levels, and 3′-untranslated regions play a major role in control of mRNA decay. We show here that trypanosomes have a homologue of Upf1, *Tb*UPF1, which interacts with *Tb*UPF2 and (in an RNA-dependent fashion) with poly(A) binding protein 1, PABP1. Introduction of a premature termination codon in either an endogenous gene or a reporter gene decreased mRNA abundance, as expected for nonsense-mediated decay, but a dependence of this effect on *Tb*UPF1 could not be demonstrated, and depletion of *Tb*UPF1 by over 95% had no effect on parasite growth or the mRNA transcriptome. Further investigations of the reporter mRNA revealed that increases in open reading frame length tended to increase mRNA abundance. In contrast, inhibition of translation, either using 5′-secondary structures or by lengthening the 5′-untranslated region, usually decreased reporter mRNA abundance. Meanwhile, changing the length of the 3′-untranslated region had no consistent effect on mRNA abundance. We suggest that in trypanosomes, translation *per se* may inhibit mRNA decay, and interactions with multiple RNA-binding proteins preclude degradation based on 3′-untranslated region length alone.

## Introduction

The eukaryotic nonsense mediated decay (NMD) pathway degrades mRNAs with mutations that result in premature termination of translation [1], [2], [3]. Premature termination codons (PTC) can occur through frame-shift or point mutations, or as a consequence of splicing defects. NMD requires translation in order to recognise a PTC, and occurs in the cytoplasm [4], [5]. An ATP-dependent RNA helicase called Upf1 is essential for NMD [6], [7], [8], [9], [10]. The helicase activity is required [11]: over-expression of a helicase-dead Upf1 mutant had a dominant-negative effect [12]. Some forms of NMD also require Upf2, which interacts with Upf1. Upf1 sequences have been found in all eukaryotic groups tested so far [13], [14]. In contrast some organisms have no obvious Upf2, and its loss is correlated with mutations in the Upf2-interaction domain of Upf1 [14], [15]. NMD in mammals involves phosphorylation of Upf1 by the Smg-1 kinase; it is not clear if the phosphorylation is necessary in yeast and Smg-1 is not conserved [16].

A nonsense codon can be recognised as a PTC by various mechanisms, depending on both the gene and the species. In several organisms, the nature of the 3′-UTR is important: NMD can be triggered by the presence of an abnormally long 3′-UTR, or by specific sequences in the 3′-UTR or around the termination codon [17]. In a recent study of human cells, Upf1 loading on several mRNAs was shown to be directly proportional to UTR length, suggesting that UPF1 was able to bind non-specifically to the parts of the mRNA that were not being actively translated and thereby “measure” the 3′-UTR length [18]. Most *Saccharomyces cerevisiae* 3′UTRs are less than 300 bases long [19] and the presence of a 3′-UTR that is longer than usual can trigger NMD [20]. Similarly, the distance from the PTC to the poly(A) tail has been shown to determine NMD in *Drosophila melanogaster* [21], [22], and long 3′-UTRs are associated with NMD in *Arabidopsis thaliana* [23]. This type of NMD depends on ribosome release factors, which can complex either with Upf1 or with poly(A) binding protein (PABP). A current model suggests that when a 3′-UTR is abnormally long, interactions with Upf1 predominate, resulting in recruitment of the mRNA decay machinery [15], [21], [24], [25]. Upf2 is not obligatory for this type of NMD in yeast [26] or human cells [15]. In *S. pombe* the determinants for NMD are uncertain, the main criterion appearing to be the ORF length [27]. *Giardia*, too, exhibits NMD-like mRNA decay, but the precise signals are unknown and dependence on UPF1 has not been demonstrated [13].

Mammalian cells have a splicing-dependent NMD pathway, which depends on the proteins of the exon junction complex (EJC), which are deposited −20 to −24 nucleotides upstream of the splice junction [2], [7], [23]. The EJC contains the NMD factor Upf3; this recruits Upf2 which in turn recruits Upf1. In contrast, in *Schizosaccharomyces pombe*, unspliced mRNAs were subject to weak NMD: the abundance of a reporter mRNA decreased concordantly with coding region length, and 3′-UTR length had little influence [27]. The presence of a nearby intron, either upstream or downstream of the PTC, enhanced NMD and the EJC was not required [27].

NMD targets mRNAs that have short open reading frames (ORFs) upstream of the start codon [28], [29], [30], suppresses splicing defects [28], [31], [32], [33] and disposes of non-coding polyadenylated RNAs [34]. To investigate the overall role of NMD in regulation of gene expression, transcriptome profiles have been obtained for cells depleted of NMD factors. In *S. cerevisiae*, nearly a tenth of all mRNAs showed increased abundance in Δ*upf1* cells, [35], [36], [37]. The identified transcript set was markedly skewed towards mRNAs that had relatively low abundances, and about half of the affected mRNAs were bound to Upf1, suggesting that they were direct NMD targets [38]. Depletion of Upf1 in animals caused 2–10 fold increases in many mRNAs, a substantial proportion of which are likely to be direct NMD substrates [31], [37], [39]. In *Caenorhabditis elegans*, for example, depletion of Upf1 increased the abundance of mRNAs that arose from splicing errors, or had upstream open reading frames [28].

Despite all this, the full physiological importance of NMD is not yet clear. Notably, Upf1, Upf2 and Upf3 are not essential for viability in yeast or *Caenorhabditis elegans*. Upf1 is essential for shoot development in *Arabidopsis* [40], mammalian embryonic viability [41] and *Drosophila* development [42]; but it is not known whether NMD-specific or non-NMD-specific function(s) of Upf1 are implicated. So far, the only protist in which NMD has been investigated is *Giardia lamblia*. In *Giardia*, introductions of PTCs into a luciferase reporter mRNA reduced mRNA abundance by up to 70% [13]. The dependence of the PTC-induced mRNA decrease on Upf1 was not tested, but extra expression of tagged Upf1 reduced the abundance of the truncated luciferase mRNA further and influenced a small number of native mRNAs [43].

Kinetoplastids are protists that diverged from animals and plants early in eukaryotic evolution. In Kinetoplastids, transcription is polycistronic. Individual mRNAs are cleaved from the precursor by *trans* splicing of a 39 nt capped leader to the 5′ end of the RNA, and by polyadenylation at the 3′-end; only one *cis*-spliced mRNA has been experimentally demonstrated [44]. The abundances of mRNAs are determined post-transcriptionally and variations in mRNA half-lives have been extensively documented. In most cases examined, mRNA abundances were shown to be determined by sequences in the 3′-UTR and regulatory RNA-binding proteins [45], [46], [47]. The mRNA decay machinery is overall similar to that in yeast, plants, and animals [48], [49], [50], [51], and RNA interference is present [52], but the existence of NMD has hitherto not been documented. In this paper we investigate the function of Upf1 and the possible existence of NMD in the Kinetoplastid *Trypanosoma brucei*.

## Results

### Sequence analysis and domain organisation of *Tb*UPF1 and *Tb*UPF2

The trypanosome genome has single loci encoding *Tb*UPF1 (Tb927.5.2140) [14] and *Tb*UPF2 (Tb11.02.4270). The *TbUPF1* homolog encodes a protein of predicted molecular weight of 93.3 kDa which shares 42.77% and 43.58% identity with human and *S. cerevisiae* Upf1, respectively (see Figure S1). It has a highly conserved N-terminal Cysteine-Histidine-rich domain (CH domain) which includes the amino acid residues needed for interacting with Upf2 [14] – present in all other sequences analysed except that from *Giardia intestinalis*. The amino acid sequence from residue 368 to 608 of *Tb*UPF1 contains the ATPase and DEAD-like helicase domains that are known to be essential for NMD.

*Tb*UPF2 is a protein of 162.5 kDa. The N-terminal part contains two MIF4G-like domains with very low scores (InterPro) followed by a single consensus MIF4G domain (SMART score 0.0013), analogous to the third MIF4G domain of human Upf2. There is then a Glu/Asp-rich acidic region. We identified no matches for Upf3, Smg-6 or Smg-7. BLASTp using Smg-1 revealed a variety of kinase domains but no clear homologue.

### Effect of *Tb*UPF1 and *Tb*UPF2 depletion on trypanosome growth

**Figure 1:**
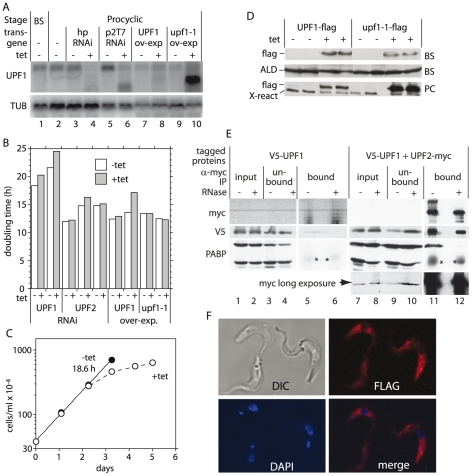
Effects of alterations in UPF1 expression on growth, interaction with UPF2, and localisation. (A) Procyclic trypanosomes with or without tetracycline-inducible *Tb*UPF1-FLAG or *Tb*upf1-1-FLAG transgenes (induced for 48 h), or inducible RNAi hp-hairpin (induced for 72 h), p2T7 – opposing T7 promoters (induced for 24 h) were grown with (+) or without (−) tetracycline (200 ng/ml) for the indicated time and then harvested for RNA preparation. Northern blots were probed for *TbUPF1* or tubulin (*TUB*) RNA. The extra band in lane 6 is the dsRNA synthesised from *TbUPF1* RNAi plasmid and the extra bands in lanes 8 and 10 are the exogenous expressed *TbUPF1/Tbupf1-1* transcripts. (B) Procyclic cells containing hp RNAi or over-expression plasmids were grown in presence and absence of tetracycline (0.1 ug/ml) and the cell count taken every 24 hours for at least 7 days. Cells were diluted to 5×10^5^ cells/ml to maintain exponential growth. Cumulative growth curves were plotted using Kaleidograph, and the doubling times for each line in the presence and absence of tetracycline were estimated using the computer-fitted growth curves. (C) Growth curve of procyclic trypanosome containing the p2T7 *UPF1* RNAi construct. Details of the growth conditions and induction are as in (B). (D) Western blot analysis on 2×10^7^ cells of bloodstream or procyclic trypanosomes expressing flag-tagged wild-type *Tb*UPF1 or the helicase mutant *Tb*upf1-1, using anti-flag antibody. The control antibody was anti-aldolase for bloodstream forms; for procyclics a cross-reacting band is shown. (E) Interaction of tagged *Tb*UPF1 with *Tb*UPF2. *Tb*UPF2-myc (lanes 7–12) was expressed in trypanosomes with a V5-tagged *TbUPF1* gene. Cells with no myc-tagged protein (lanes 1–6) served as a control. Extracts were immunoprecipitated with anti-myc, in the presence or absence of RNase. 2.5% of the input (lanes 1, 2, 7, 8) and unbound (lanes 3, 4, 9, 10,) fractions, and the whole eluates (lanes 5, 6, 11, 12,) were separated by SDS-PAGE, blotted, and probed with antibodies to myc, V5 and PABP. *IgG heavy chain. (F) *Tb*UPF1-flag is in the cytoplasm. Fixed procyclic trypanosomes expressing UPF1-FLAG were stained for the flag tag and counterstained for DNA with DAPI. Cells with no FLAG construct showed only much fainter background fluorescence.

To find out whether *Tb*UPF1 and *Tb*UPF2 were required for trypanosome growth, we created bloodstream and procyclic trypanosome cell lines with tetracycline-inducible RNAi. We failed to generate an appropriate anti-UPF1 antibody, so to assess the effect of the RNAi on the protein, we tagged one copy of *UPF1 in situ*, so that the expressed protein had an N-terminal V5 tag. Two procyclic form clones with up to 88% reduction in the mRNA (Figure 1A) showed slightly prolonged division times but this was independent of the presence of tetracycline (Figure 1B), so might be due to clonal variation. A procyclic line in which dsRNA against *TbUPF1* was made by synthesising the entire ORF as a double strand, using opposing T7 polymerase promoters, grew slowly in the absence of tetracycline and almost completely stopped dividing upon tetracycline addition (Figure 1C). This could have been a consequence of extremely effective depletion, but off-target effects cannot be ruled out. In bloodstream trypanosomes with one V5-tagged *UPF1* allele, RNAi targeting *UPF1* resulted in over 95% depletion of V5-UPF1 without any effect on growth (not shown). Inducible RNAi against *TbUPF2* did not affect procyclic trypanosome growth or morphology up to 7 days post-tetracycline addition (Figure 1B) although the transcript was 80% reduced after 3 days (not shown). In a recently published high-throughput RNAi screen, cells with *UPF1* or *UPF2* RNAi even appeared to have a selective advantage in bloodstream forms, but not procyclic forms [53]: although high-throughput results must always be viewed with caution, in this case they were consistent with the results of our focussed experiments. All evidence so far therefore indicates that neither UPF1 nor UPF2 is required for bloodstream trypanosome growth and division, but there were hints that some UPF1 might be needed for optimal growth of procyclic forms. We do not know whether either protein is completely dispensable because a knockout was not attempted.

For over-expression analysis, we created bloodstream and procyclic trypanosomes with tetracycline-inducible expression of FLAG-tagged wild type *Tb*UPF1. We also inducibly expressed an R747C mutant version, *Tb*upf1-1. This is equivalent to the yeast R779C and human R844C Upf1 mutants, expression of which abrogated NMD in a dominant-negative fashion [12], [54]. Expression of either protein in bloodstream forms had no effect on growth. In procyclic forms, the *TbUPF1*-flag and *Tbupf1-1*-flag transcripts were, respectively, <2-fold and 13-fold more abundant than the endogenous *TbUPF1* transcript (Figure 1A). Correspondingly, *Tb*upf1-1-flag protein was considerably more abundant than *Tb*UPF1-flag (Figure 1D). Expression of *Tb*UPF1-flag marginally inhibited cell growth; expression of *Tbupf1-1*-flag resulted in some slightly abnormal parasite morphology (not shown) but did not change the division time (Figure 1B). The inducibly-expressed *Tb*UPF1-flag was readily detectable by immunofluorescence and located predominantly in the cytoplasm (Figure 1F).

### *Tb*UPF1 interacts with *Tb*UPF2

To find out whether *Tb*UPF1 and *Tb*UPF2 interact, we co-expressed *Tb*UPF2-myc in cells with *in situ* V5-tagged *Tb*UPF1 (Figure 1E). Immunoprecipitation with anti-myc antibody resulted in co-precipitation of both V5-*Tb*UPF1 and PABP (lane 11). The interaction between V5-*Tb*UPF1 and *Tb*UPF2-myc was not affected by RNase treatment, whereas PABP was released (lane 12). Comparison of the band intensities in lanes 7 and 8 (2.5% of input) and pull down (100%) indicated that only a small proportion of V5-*Tb*UPF1 was bound to *Tb*UPF2-myc, but the myc pull-down also showed poor efficiency (compare lanes 9 & 10 with lanes 7 & 8). The control immunoprecipitation (using cells that did not express a myc-tagged protein) pulled down very little V5-*Tb*UPF1 and no detectable PABP (lanes 5 & 6). These results confirmed that Tb11.02.4270 is indeed likely to be *Tb*UPF2, as predicted in [55]. The results also indicated that tagged *Tb*UPF2 and *Tb*UPF1 were bound to PABP in an RNA-dependent fashion.

So far, our results were consistent with a role of *Tb*UPF1 in cytoplasmic mRNA metabolism.

### The abundance of a PTC-containing trypanosome mRNA decreases with ORF length

**Figure 2:**
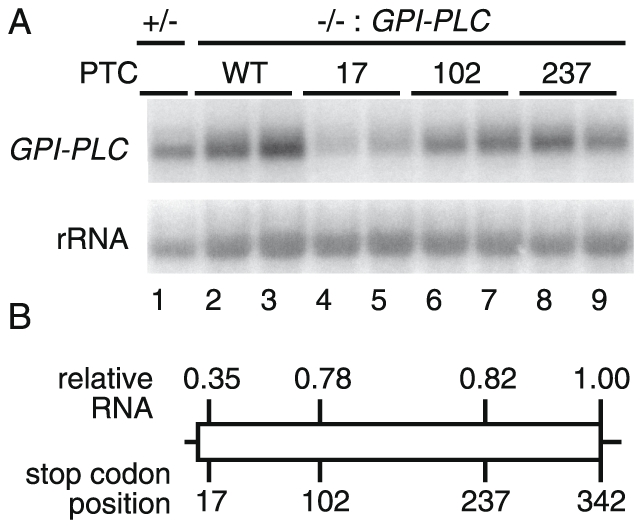
Shortening the *GPI-PLC* ORF length reduces mRNA abundance. (A) Northern blots of RNA extracted from two independent clones containing *GPI-PLC* transgenes, wild type (342 codons, lanes 2,3) or with PTCs at codons 17 (lanes 4,5) or 102 (lanes 6,7) or 237 (lanes 8,9). RNA extracted from a GPI-PLC +/− cell line is shown for comparison in lane 1. (B) Quantitation of *GPI-PLC* mRNA by phosphorimager, plotted onto a map of the open reading frame. The values are averages of measurements from the two clones and are shown relative to the wild type transgene, after normalization for loading using rRNA. The *GPI-PLC* +/− cells gave a relative mRNA abundance of 0.91.

To find out whether trypanosomes degrade mRNAs containing premature termination codons (PTCs) we inserted PTCs into a trypanosome ORF. We started with bloodstream trypanosomes lacking the non-essential gene (Tb927.2.6000) encoding glycosyl phosphatidylinositol phospholipase C (*GPI-PLC*) [56]. We took plasmids containing either the wild-type *GPI-PLC* gene or a PTC-containing version, with an associated selectable marker. These plasmids were transfected into trypanosomes so that they would integrated into the original locus [57] (Figure S2). The wild-type open reading frame contains 342 codons; the modified transgenes contained PTCs at codon 17, codon 102 or codon 237. We had previously reported that the PTC at position 17 (51 nt ORF) had little effect on the amount of mRNA, but the abundance had not been quantified [57]. More careful analysis revealed that this PTC reduced the steady state level of the *GPI-PLC* mRNA to 35% of normal (Figure 2). PTCs at codons 102 or 237 reduced the levels marginally, to ∼80% of normal. These cells lines contain most of the drug resistance markers available in trypanosomes. To determine the role of *Tb*UPF1 in the *GPI-PLC* mRNA decay, we attempted to deplete *TbUPF1* mRNA by transient transfection of morpholino oligonucleotides; this had little effect but the depletion was insufficiently effective to draw any conclusions.

### The abundance of a PTC-containing reporter mRNA decreases concordantly with ORF length

**Figure 3:**
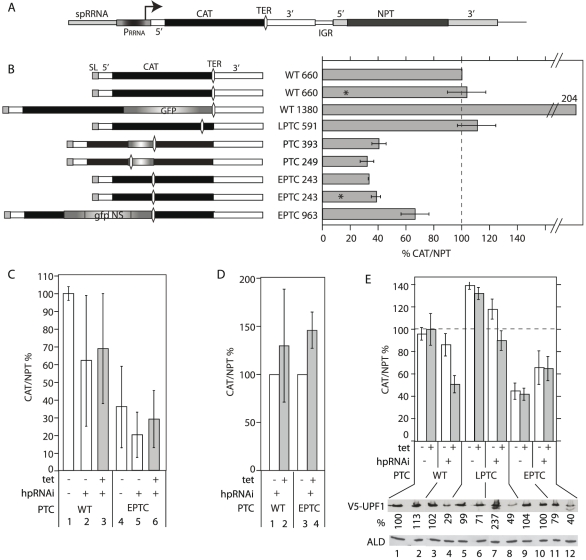
Effect of ORF length on mRNA levels from a *CAT* reporter. (A) Map of the *CAT-NEO* reporter plasmid used to analyse requirements for NMD. *spRRNA*: non-transcribed portion of rRNA spacer, used for integration into the genome; P_RRNA_ – rRNA promoter; 5′: 5′-UTR; 3′: 3′-UTR; TER – symbol for termination codon. This plasmid was used in procyclic forms. For bloodstream forms, *spRRNA* and P_RRNA_ were replaced with a segment from the tubulin locus. (B) Effect of the position of the termination codon on the *CAT/NPT* ratio in procyclic trypanosomes, with pol I transcription of the reporter. The cartoons on the left illustrate the *CAT* mRNAs investigated. SL: spliced leader; GFP: complete or partial GFP coding region. The 5′-UTR of the WT mRNA is 70 nt, the *CAT* ORF 660 nt and the 3′-UTR 326 nt. The graphs on the right show the ratio of *CAT* to *NPT* mRNA, normalised to wild-type, expressed as a percentage. Results are arithmetic mean and standard deviation for at least three biological replicates. Asterisks indicate cloned cell lines, the rest of the results are for populations. (C) Effect of ORF length and UPF1 RNAi on *CAT/NPT* mRNA ratio in bloodstream trypanosomes, with pol II transcription of the reporter. Results are for 3–4 independent cloned lines, each measured once or twice, and are expressed as arithmetic mean ± standard deviation. The arithmetic mean *CAT/NPT* ratio for *WT-CAT* was set to 100%. Columns 2,3 and 5,6 are for cell lines containing a hairpin *UPF1* RNAi plasmid, without (2, 5) or with (3, 6) tetracycline. (D) Results are the same as in (C), but each cell line was considered individually, with the *CAT/NPT* value minus tetracycline set to 100%. (E) Effect of *TbUPF1* RNAi on levels of *CAT* mRNAs in procyclic forms. Trypanosomes with *in situ* V5-tagged UPF1 were transfected with reporter plasmids and the hp*TbUPF1* RNAi plasmid. Tetracycline was added where indicated, for 48 h. The ratio of *CAT* to *NPT* mRNA was measured by real-time PCR, and expressed as a percentage of wild-type. Results are arithmetic mean and standard deviation for at least three biological replicates. The Western blots beneath the graph show expression of V5-UPF1, with aldolase (ALD) as loading control. The V5 signals were normalised using the aldolase band, and expressed as a percentage relative to lane 1. The high V5 signal in the LPTC RNAi line might be due to tagging of both alleles. The double band of V5-*Tb*UPF1 was rarely seen; it might be caused by phosphorylation [79].

To test for NMD in procyclic-form *T. brucei*, we used a reporter containing the chloramphenicol acetyl transferase (*CAT*) ORF followed by the 3′-UTR of the actin gene (*CAT-ACT* construct). Downstream of this was the neomycin phosphotransferase (*NEO*) gene, with 5′ and 3′-UTRs (Figure 3A). We transfected linearized plasmid into procyclic trypanosomes, and selected for G418 resistance. The plasmid was designed to integrate into the silent rDNA spacer, with an rRNA promoter driving transcription by RNA polymerase I. Using this system, the level of *CAT* mRNA could be measured using *NEO* RNA as an internal control for both transcription rate and plasmid copy number. Use of pol I gave us sufficient RNA for quantification and detection on Northern blots, enabling us to confirm that mRNAs had the correct size (shown later). We note that in trypanosomes, any effect of *trans* splicing on mRNA decay should be seen equally in mRNAs made by RNA polymerase I or RNA polymerase II, since the evidence so far indicates that transcripts made by either polymerase are processed with equal efficiency [58]. All measurements were made in cells that had been growing for at least 2 days without G418.

The *CAT* ORF is of prokaryotic origin. To find out if a nonsense-mediated decay effect could be seen, we inserted PTCs at various positions. The *CAT* termination codon is at nt 660 (*WT-CAT*, WT660 in Figure 3B). Insertion of 4 nt at position 218 resulted in a frame shift, giving a TGA termination codon at position 243 and a second TAG 5 codons downstream. This construct was named “early PTC” (*EPTC-CAT*) and yielded no detectable CAT protein (judged by Western blotting, not shown). Translation reinititation is unlilkely with this construct since the next ATG after the PTC is 45 nt downstream. A similar insertion at position 519 gave a TGA at position 591 (late PTC, *LPTC-CAT*) (Figure 3B); in this case two more in-frame termination codons are 9 and 10 codons downstream, with two ATGs within 18 nt of the PTC; should any translation re-initiation occur, the longest possible ORF would extend only 54 nt beyond the *WT*-*CAT* termination codon.

In addition to the *EPTC-CAT* and *LPTC-CAT* ORFs, we inserted segments of the *GFP* gene at various positions to give ORF lengths of 393, 963 and 1380 nt. Cell lines were generated and we confirmed that the mRNA sizes were as expected (not shown). We then measured the level of *CAT* mRNA by reverse transcription and real-time PCR (Figure 3B). ORFs of 243–393 nt (EPTC243, EPTC249, EPTC393 in the Figure) all yielded 30–40% of wild-type RNA, while the 1380 nt *CAT-GFP* ORF doubled the amount of mRNA relative to *WT-CAT*. Taken together these results were consistent with three hypotheses: either the introduction of a PTC *per se* decreased mRNA abundance, or the length of the ORF or the 3′-UTR was the determining factor. To follow this up, we made a construct with a 963 nt *CAT-GFP* ORF, but the same 3′-UTR as the *EPTC-CAT* mRNA. The resulting *EPTC-CAT-GFP* mRNA level was (65% of WT) intermediate between that of *EPTC* and *WT-CAT*. This suggests that long ORFs favour mRNA abundance, while the presence of the untranslated *CAT* segment has a negative influence. The results do not show whether the effect of the untranslated *CAT* segment is sequence-specific, or is related to the overall length of the 3′-UTR.

To measure mRNA half-lives, we treated the cells with Sinefungin and Actinomycin D to halt transcription, and then measured mRNA levels by Northern blot. We consistently observed, as seen previously for experiments using Actinomycin D alone [59], that the apparent mRNA levels either stayed constant, or increased over the first 2 h after inhibitor addition (Figure S3A), so in general we calculated half-lives starting from the 2 h time-point. Pooling results for all cell lines with normal levels of UPF1 and UPF2 (including RNAi lines with no tetracycline induction, a total of at least 8 measurements for each CAT construct), we found that the half-lives of the *WT-CAT* and *EPTC-CAT* mRNAs *were* 2.4±0.2 h and 1.8±0.5 h respectively. The result for *WT-CAT* agrees with the previous estimate made using Actinomycin D alone [60]. The half-life numbers were, however, so strongly dependent on which time points were used to plot the decay curves that it was impossible to make any quantitative judgements from the results.

To make sure that the results with the reporter were not artefacts of pol I transcription, and to find out whether PTC introduction affected mRNA abundance in bloodstream forms, we integrated the *WT-CAT* and *EPTC-CAT* ORFs into the tubulin locus in bloodstream-form trypanosomes. Results from four independent cell lines yielded *EPTC-CAT* mRNA levels of between 8% and 59% relative to full-length *CAT* (Figure 3D, compare bars 1 and 4). This shows that the effect of the PTC was independent of both the transcribing polymerase and the trypanosome life-cycle stage.

### Effect of RNAi targeting *Tb*UPF1 on reporter mRNAs

We now investigated whether *Tb*UPF1 was required for the relatively low abundance of the *EPTC*-*CAT* mRNA. We made cell lines that expressed *WT-CAT*, *LPTC-CAT* or *EPTC-CAT* mRNA and had inducible *TbUPF1* RNAi. The levels of *CAT* mRNA were measured with or without induction of RNAi. For bloodstream forms, expressing *WT-CAT*, using RNA polymerase II, we assayed 3 independent cloned lines. The *CAT* mRNA levels in the RNAi lines showed considerable variation between clones (Figure 3C, lanes 2,3,5,6). To measure the effect of RNAi, we therefore compared individual clones with and without tetracycline. For three *EPTC*-*CAT* lines, the increase in *CAT* mRNA was 1.3–1.7 fold; however similar increases were seen in 3 out of 4 *WT*-*CAT* lines (RNA levels changed 0.5–1.9 fold) (Figure 3D). Thus depletion of UPF1 did not specifically reverse the effect of the PTC on reporter mRNA abundance. Preliminary results for *UPF2* RNAi revealed no effect at all on either mRNA (not shown).

In the procyclic cell line with the *WT-CAT* gene (polymerase I transcription) and the *TbUPF1* RNAi plasmid, *TbUPF1* RNAi caused a 30–40% decrease in *WT*-*CAT* mRNA abundance (Figure 3E, lanes 3 & 4). With the *LPTC-CAT* transgene, results were similar (lanes 7 & 8). Addition of tetracycline to cells with no RNAi construct had no effect (Figure 3E, lanes 2, 6 and 10). In the cell line with the *EPTC*-*CAT* transgene, the increase in *CAT* mRNA was unaffected by RNAi induction (Figure 3D lanes 11 &12). From all experiments, so far we therefore concluded that introduction of PTCs – or decreasing the ORF length - reduced the abundances of the *CAT* and *GPI-PLC* mRNAs in trypanosomes, but there was no convincing evidence for a role of UPF1 in destruction of *EPTC-CAT* mRNA.

### The length of the 3′-UTR does not reproducibly affect reporter mRNA abundance

**Figure 4:**
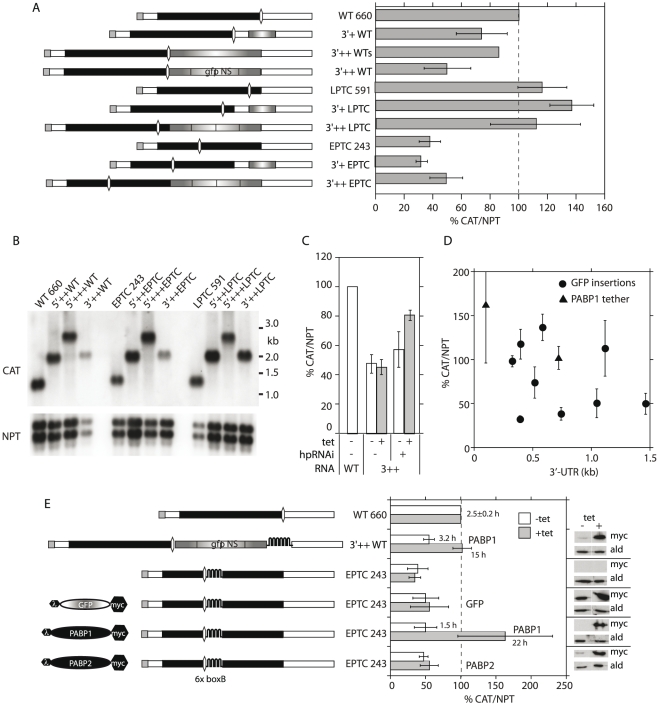
Effect of the 3′-UTR length and PABP on *CAT* mRNA abundance. (A) Effect of the 3′-UTR length on the *CAT/NPT* ratio. The cartoons on the left illustrate the *CAT* mRNAs investigated. *gfpNS*: GFP coding region with no start codons. Details as in Figure 3. The sizes of the mRNAs were verified by Northern blotting (not shown). (B) Northern blot showing the sizes of selected reporter mRNAs. (C) Effect of *UPF1* RNAi on the steady-state level of the 3′++WT mRNA. Results are mean and standard deviation of 3 independent experiments. (D) Relationship between 3′-UTR length (or termination codon – PABP distance) and mRNA abundance using data from (A) and (E). Results are mean ± standard deviation. (E) Tethering of PABP1 and PABP2. Six copies of BoxB were inserted either between the EPTC *CAT* cassette and the 3′-UTR in the EPTC 243 construct (Figure 3B), or after the AUG-less GFP coding sequence in the 3′-UTR of the 3′++WT construct (Figure 4A). Cell lines containing the various *CAT* mRNAs (as indicated to the left of the bar graph) were also transfected with tetracycline inducible plasmids expressing proteins bearing an N-terminal lambda-N peptide, and a C-terminal myc tag. Proteins were GFP, PABP1 (Tb09.211.0930) or PABP2 (Tb09.211.2150), shown to the left of the mRNA cartoons. Expression of the tagged proteins was induced for 48 h using 100 ng/ml tetracycline. The Western panels on the right, which were probed with antibody to the myc tag and to aldolase (control) come from a single experiment and exposed film. The amounts of *CAT* mRNA relative to *NEO*, measured by qPCR, are shown as mean and standard deviation for three independent experiments.

Our results in Figure 2 and 3B were consistent with the prediction that mRNA degradation increases with the distance of the termination codon from the poly(A) tail. To find out whether 3′-UTR length really was important, we extended the 3′UTR of the *WT-CAT* mRNA and measured the relative mRNA abundance, using procyclic trypanosomes. Inserting 190 nt of *GFP* sequence (3′+WT), or even a complete *GFP* ORF (720 nt, 3′++WTs) had no effect on *WT-CAT* mRNA levels (Figure 4A). Correct processing of the mRNA was confirmed by Northern blotting (Figure 4B).

Since the 3′-UTR *GFP* insertion was immediately downstream of the *WT-CAT* termination codon, potentially allowing reinitiation, we created a new plasmid in which the 720 nt *GFP* ORF had been mutated so that it lacked initiation codons (*GFPns*, GFP-no-start). The abundance of mRNA (3′++WT) from this plasmid was 55% of WT, and the effect was reversed to 82% by RNAi against *TbUPF1* (Figure 4C). This was consistent with 3′-UTR-dependent and UPF1-dependent NMD. We were, however, unable to confirm this result with the *LPTC-CAT* and *EPTC-CAT* ORFs. Lengthening their 3′UTRs by 720 nt, using the ATG-less GFP (from 393 to 1112 nt in *LPTC-CAT* and 743 to 1463 nt in *EPTC-CAT*) did not decrease *CAT* mRNA levels (Figure 4A); for *EPTC-CAT*, results stayed between 35% and 40% even with the longest extension. When we put together the results of all these reporter experiments, the length of the 3′-UTR, over a 330 nt–1.2 kb range, had no consistent effect on trypanosome mRNA abundance (Figure 4D). If there is a 3′-UTR length effect at all, it must be overridden even by minor differences in the 3′-UTR sequence.

### Tethering of poly(A)-binding protein inhibits trypanosome mRNA decay

In mammals, budding yeast and *Drosophila*, tethering of cytoplasmic poly(A)-binding protein (PABP) downstream of a PTC inhibits NMD [61], [62]. It was thought that the tethered PABP specifically antagonised NMD by effectively shortening the 3′-UTR [61], but recently the effect has been shown to be more general and not dependent on PTC recognition [62]. To find out whether the PABP effect was seen in trypanosomes, we inserted six “B” boxes downstream of *EPTC-CAT*, or downstream of GFPns in the 3′++WT construct (Figure 4E). This lengthened each 3′-UTR by 270 nt but had no effect on mRNA abundance (compare Figures 4A and 4E). Proteins bearing a lambda N peptide at the N-terminus, and a myc tag at the C-terminus, were inducibly expressed in cell lines expressing the new mRNAs and the effect on *CAT* mRNA was measured. All three proteins were expressed at comparable levels. There are two PABPs in trypanosomes, PABP1 (Tb09.211.0930) and PABP2 (Tb09.211.2150). Tethering of either GFP or PABP2 to the *EPTC-CAT*-box B mRNA had no effect on mRNA abundance. In contrast, tethering of PABP1 to either 3′++WT or *EPTC-CAT* mRNA – decreasing the stop codon -PABP distances to 720 and 100 nt respectively - indeed increased mRNA levels. PABP1 tethering caused complete stabilization of the mRNAs (Figure 4E and Figure S3G, H).

### Changing the 5′-UTR length decreases mRNA abundance and inhibits translation

**Figure 5:**
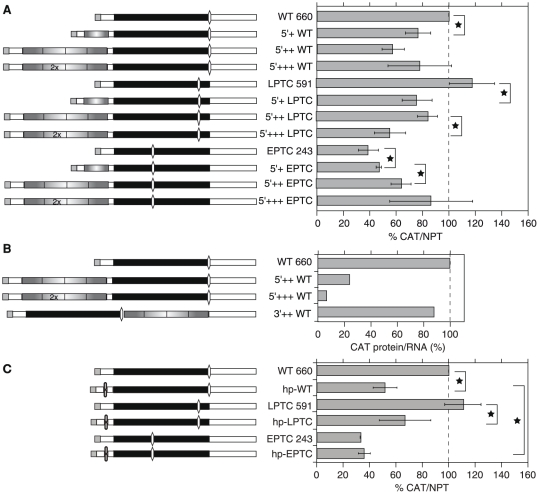
Role of the 5′-UTR length and translation. (A) Effect of the 5′-UTR length on the *CAT/NPT* ratio. Stars indicate where paired values are different at the P = 0.05 level or below (Students t-test, two-tailed, unpaired). The sizes of the mRNAs were verified by Northern blotting (not shown). Details as in Figure 3. (B) Effect of the 5′-UTR length on the amount of CAT protein. The amount of CAT protein was measured relative to aldolase (loading control). Results are the average of duplicate measurements, variation was very low. (C) Effect of the translation inhibition length on the *CAT/NPT* ratio. The double ovals in the 5′-UTRs represent hairpins which completely inhibit translation. Other details are as for Figure 4A.

We next wanted to check whether the physical length of the ORF or the distance from the 5′-end to the stop codon, influenced mRNA abundance. We therefore extended the 5′-UTRs of *WT-CAT*, *LPTC-CAT* and *EPTC-CAT* by inserting ATG-less *GFP* sequences of 190 nt (5′+), 720 nt (5′++) or 1440 nt (5′+++). Results are shown in Figure 5A and Northern blots to verify RNA sizes are in Figure 4B. Lengthening the 5′-UTR in front of the *WT-CAT* or *LPTC-CAT* ORFs decreased the mRNA levels by 20–50%. In contrast, the amount of CAT mRNA from the *EPTC-CAT* constructs increased steadily as the length of the 5′-UTR increased; with the longest 5′-UTR, rescue was complete, since the 5′++EPTC mRNA level was the same as that of the 5′++WT mRNA. Thus the level of mRNA was *not* consistently increased in parallel with the distance of the termination codon from the 5′-end.

NMD depends on the recognition of the nonsense mutations by the translation machinery. It was therefore important to examine the effects of 5′-UTR extension on translation. To do this, using *WT-CAT* cell lines, we measured amounts of CAT protein by Western blotting, then normalised them to the amount of *CAT* mRNA from Figure 5A. Insertion of *GFPns* in the 5′-UTR strongly inhibited translation whereas the same sequence in the 3′-UTR did not (Figure 5B). Thus we could not see whether the effects we had seen were due to 5′-UTR length per se, or to translation inhibition.

To distinguish the effects of translation inhibition from those related to 5′-UTR length, we sought a method to inhibit translation alone. Treatment of trypanosomes with protein synthesis inhibitors increases the levels of many mRNAs – including the *WT-CAT* mRNA used in this study (not shown). The reasons have not been defined, but a requirement for unstable protein factors for mRNA decay (e.g. [57]), or effects on mRNA processing [63] have been suggested (reviewed in [48]). To analyse translation alone, we therefore inserted a translation-inhibitory hairpin into the 5′-UTRs of the reporter plasmids (control data for translation shown later). This decreased the abundances of the *WT-CAT* and *LPTC-CAT* mRNAs (Figure 5C) in the same way as lengthening the 5′-UTR (Figure 5A). This indicates that translation favours mRNA maintenance. In contrast, the amount of *hp-EPTC-CAT* mRNA was similar to that of *EPTC-CAT* mRNA (Figure 5C). The simplest interpretation of this result is that *EPTC-CAT* mRNA has low abundance because of NMD, while the *hp-EPTC-CAT* and *hp-CAT* mRNAs have low abundance because they have no ribosomes on the ORF.

### Tethering of UPF1 to a 3′-UTR can decrease mRNA abundance

**Figure 6:**
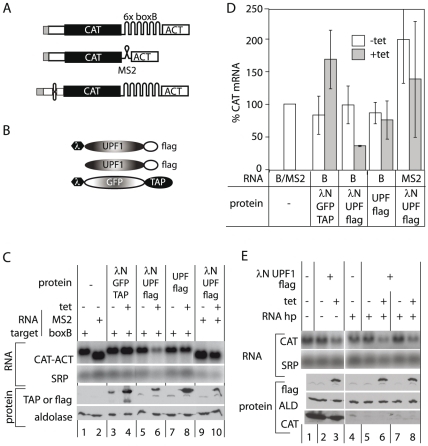
Tethering of *Tb*UPF1 decreases the abundance of *CAT-ACT* mRNA. (A) Reporter mRNAs with *ACT* 3′-UTRs. used for tethering experiments, not to scale. For details see text and Table 1. The mRNAs were produced from a T7 promoter, in cells expressing T7 polymerase. (B) Fusion proteins used for tethering. For details see text and Table 1. (C) Northern blot showing *CAT-ACT* mRNAs in cells inducibly expressing GFP and *Tb*UPF1 fusion proteins. Appropriate Western blots are beneath. Expression of tagged *Tb*UPF1 or GFP was induced with 1 µg/ml tetracycline for 2 days. A portion of the cells was used for western analysis and the rest was used for total RNA isolation. Northern blots were probed with full length *CAT* probe and *SRP* was used as loading control. For western analysis, cell lines expressing the *Tb*UPF1 with a flag tag were probed with anti-flag antibody and those with the TAP tag were probed with PAP antibody. The same blots were probed with anti-aldolase antibody as a loading control. (D) Quantitation of the data in (C). *CAT* mRNA amounts are expressed as the percentage of the appropriate control - lane 1 in (C). Results are the arithmetic mean of and standard deviation for at least three biological replicates or cell lines. (E) As for (C), but including cells expressing *hp-CAT-B-ACT*.

To find out whether UPF1 was capable of affecting mRNA levels at all, we used a tethering approach. As reporter, we used the *CAT-ACT* mRNA, with either 6 copies of the B box sequence, or two copies of MS2 stem-loop downstream of the stop codon (Figure 6A). Stable clonal cell lines were obtained that expressed the reporter mRNAs constitutively from the T7 promoter. These reporter lines were transfected with the plasmids giving inducible expression of lambdaN-tagged protein constructs: lambdaN-UPF1-flag (UPF1 with a C-terminal FLAG tag), lambdaN-GFP-TAP (Green fluorescent protein with a TAP tag) or UPF1-flag without the lambdaN (Figure 6B). A representative set of results obtained with the *CAT-ACT* 3′UTR mRNA is shown in Figure 6C and quantitation for 3 biological replicates in Figure 6D. A reproducible decrease of the reporter mRNA was seen upon induction of lambdaN-UPF1-FLAG in the *CAT-B-ACT* cell line (Figure 6C&D, lanes 5,6). This decrease was dependent on the RNA-protein interaction because expression of lambdaN-UPF1-FLAG (Figure 6C&D, lanes 9,10) did not decrease the amount of *CAT-MS2-ACT* mRNA, Expression of UPF1-FLAG (Figure 6C&D, lanes 7,8) and tethering of lambdaN-GFP-TAP (Figure 6C&D, lanes 3,4) also did not decrease *CAT-B-ACT* mRNA. Results suggested that the UPF1 tethering was indeed affecting mRNA degradation (see Figure S4). Tethering of lambdaN-UPF1-FLAG to a target mRNA with a translation-inhibitory hairpin also caused a decrease in target mRNA (Figure 6E, lanes 5–8). Preliminary results (not shown) indicated that tethering of mutant upf1-1 also caused degradation. Overall, this would imply that *Tb*UPF1 may be capable of recruiting the degradation machinery directly via protein-protein interactions.

### Effects of ORF and UTR lengths across the transcriptome

To find out whether depletion of *Tb*UPF1 had any effect on the steady-state transcriptome, we did microarray experiments using procyclic trypanosomes with *TbUPF1* RNAi (full-length RNAi construct). No changes above two-fold were found, so the results have not been submitted to a database. Since a few probes showed up-regulation of 1.3–1.6-fold, we chose one - Tb927.10.12900 (up 1.54-fold) - for further analysis. Northern blotting revealed two bands:, one was the expected monocistronic RNA of 0.9 kb (increased 1.3-fold by *UPF1* RNAi) and another of 4.5 kb that was increased 2.4-fold by *UPF1* RNAi (Figure S5 A,B, average of two independent measurements). The origin of the 4.5 kb band is not clear (see Figure S5, legend); it may be a dicistronic transcript. We also examined the *EP* procyclin locus. This produces two RNAs which should be targeted by classical NMD, and one of these accumulated 1.3–1.5-fold upon UPF1 depletion (Figure S5C, D). Thus although UPF1 might play some role in control of aberrantly processed mRNAs, the only effects detected were marginal.

## Discussion

This paper includes some results that support the existence of a classical NMD pathway in *T. brucei*. There are genes encoding UPF1 and UPF2, and the two proteins interact; also, tethering of UPF1 to an mRNA decreased its abundance and stability. The abundances of an endogenous mRNA and a reporter mRNA were decreased by introduction of a PTC. In a reporter mRNA with a full-length *CAT* ORF, extension of the 3′-untranslated region caused a decrease in reporter mRNA abundance which was abrogated by RNAi targeting UPF1. Many other results, however, do not support the existence of classical NMD – notably, several failures to specifically reverse apparent NMD effects by *UPF1* RNAi.

The decreases in mRNAs upon introduction of PTCs were analogous to those that have been seen in other organisms. For *GPI-PLC*, a termination codon at codon 17 reduced RNA to 35% of normal, but one at codon 102 only to 80%. For *CAT*, in contrast, a PTC at codon 81 reduced mRNA to about 35%, and again, early PTCs depressed mRNA levels more than later ones. These values are consistent with NMD in Opisthokonts. For example, PTCs at codon 83 in the *Drosophila* ADH gene reduced mRNA to 25–30% [64] and mammalian intron-dependent NMD (codon 46) depressed mRNA to 9% [65]. In *S. pombe*, intron-independent decay reduced mRNA to 43% at codon 27 and 83% at codon 140, while intron-dependent decay resulted in levels around 20% [27].

In Opisthokonts, mechanisms for PTC recognition are predominantly based on (1) the termination codon sequence context; (2) the positions of *cis* splicing junctions (marked by the EJC); and (3) the length of the 3′-UTR (as judged by the distance between the termination codon and poly(A) binding protein). In trypanosomes, option (1) seems unlikely to be important for the *CAT* reporters: the relevant region was of prokaryotic origin. Any mechanism depending on recognition of the splice junction via the EJC is unlikely. Some of the EJC proteins are present, and form a complex, in trypanosomes [66], though neither binding to the exon junction nor a role in *trans* splicing have been confirmed. Even assuming that the EJC is deposited at the *trans* splice junction, it would be stripped off by the scanning small ribosomal subunit complex before translation initiation. An involvement of the *trans* splice site *per se* cannot, however, be ruled out. Splicing-dependent, EJC-independent NMD was shown in *S. pombe* [27]: notably, NMD was stimulated by the presence of an intron *upstream* of a PTC – as would occur in trypanosomes. *S. pombe* splicing-dependent NMD was however stronger than the NMD-like effects that we saw in trypanosomes.

In a recent study of mRNP composition, UPF1 was found in all mRNPs examined, but the amount was greater in mRNAs that are susceptible to NMD, and also correlated positively with the length of the 3′-UTR [18]. In addition, even low amounts of translational read-through or reinitiation reduced NMD. Our experiments with reporters yielded very mixed evidence concerning a role of the 3′-UTR length in determining trypanosome mRNA abundance. For the *WT*-*CAT* ORF, we saw a clear decrease in *CAT* mRNA abundance after insertion of an ATG-less *GFP* sequence in the 3′-UTR. The result was, however, contradicted using the *LPTC-CAT* ORF, where the same insertion had no effect (Figure 4D). The most recent evidence suggests that at a global level, 3′-UTR length negatively affects mRNA half-lives in mouse cells [67], but the effect is both variable and weak. In trypanosomes, deep sequencing data reveals no such correlation (T. Manful, A Fadda and C. Clayton, ZMBH, manuscript submitted). This is not surprising. Trypanosome (and mouse) 3′-UTRs are somewhat longer than those of yeast: for trypansoomes, the median length is 400 nt, and 3′-UTRs over 1 kb are by no means unusual [68]. The heavy reliance of trypanosomes on regulation of mRNA stability to control gene expression may have driven the evolution of long 3′-UTRs: these will have space for multiple elements which can be recognized by regulatory sequence- or secondary-structure-specific RNA-binding proteins.

Tethering of PABP to reporter mRNAs had a very strong stabilizing effect. This is may well be unrelated to NMD - in yeast, even a non-translated mRNA can be stabilized by tethering of PABP [62]; 5′ decapping is inhibited. It was intriguing that we saw effects of PABP1, but not PABP2. In the related parasite *Leishmania*, both proteins are cytoplasmic and can associate with eIF4G in vitro, but using cytoplasmic extracts, only PABP1 was shown to copurify with eIF4G [69]. It is therefore quite possible that in trypanosomes, also, tethered PABP1 inhibits 5′-3′ degradation by binding to the cap-bound translation initiation complex. Whether this also stimulates translation, as in yeast [70] remains to be determined.

Our results leave open the question of the function of UPF1 in trypanosomes. It is likely to play some role in Kinetoplastids since the gene is well conserved throughout Kinetoplastids. *Tb*UPF1 must also be bound to some RNAs, since it was found to interact not only with UPF2, but also, in an RNA-dependent fashion, with PABP. In addition, tethering of UPF1 to an mRNA did increase the degradation rate, and there were various RNAi effects on reporters that appeared to be independent of the presence of a PTC. Depletion of *Tb*UPF1 did not affect the *T. brucei* steady-state mRNA transcriptome as judged by microarray analysis; preliminary results from an RNA-Seq study confirm this (T. Manful, ZMBH, unpublished). There was also little evidence for a requirement of either UPF1 or UPF2 in trypanosome growth and division. It is therefore possible that UPF1 affects mRNA in conditions or life-cycle stages that were not examined in our study, or has other functions.

Overall it is not clear that classical NMD exists in trypanosomes. It would not be required for disposal of pseudogene mRNAs: most trypanosome pseudogenes are either located in telomeric regions that are not transcribed, or produce transposon-related RNAs that are disposed of by the RNAi machinery [71], [72]. Alternative *trans* splicing can definitely create short upstream ORFs and bicistronic mRNAs, but their low abundance could easily be caused simply by low utilization of the alternative processing sites.

One more set of observations is worthy of comment: inhibition of translation, either by adding a secondary structure, or increasing the length of the 5′-UTR, decreased the amount of reporter mRNAs. Although recent results from yeast have shown that loss of ribosomes is not an obligatory prerequisite for mRNA decay [73], translation initiation certainly must stop before 5′-3′ degradation can begin. Both our results, and those from *S. pombe* [27] would be consistent with a link between the average number of ribosomes on an mRNA and its stability. Assuming that initiation is to some degree stochastic, the lower the average number of ribosomes on an ORF is, the higher the proportion of mRNAs with no ribosomes at all. Our observations therefore fit with the idea that release of an mRNA from polysomes can render it more susceptible to degradation.

## Methods

### Trypanosomes, cell culture, and plasmid construction

Procyclic and bloodstream trypanosomes of the Lister 427 strain, and bloodstream-form parasites of the AnTat 1.1 strain, were cultured according to standard protocols. Procyclic trypanosomes were diluted to a density of 5×10^5^/ml before the density reached 8×10^6^/ml; bloodstream cells were diluted to 1×10^5^/ml before the density reached 1×10^6^/ml. Unless noted, experiments were done using cells expressing the *Tet* repressor from pHD1313 [74]. In some cases the cells also expressed T7 polymerase. Tetracycline-dependent transcription by RNA polymerase 1 or the T7 polymerase was induced by addition of 0.1–1 µg/ml tetracycline. Selecting antibiotics were always removed at least one day before experiments were initiated.

All plasmid constructs are described in Table S1 and the oligonucleotides in Table S2. For RNA interference, dsRNA was either expressed as a stem-loop, transfected into the standard line, or using opposing T7 promoters with a line expressing T7 polymerase in addition to the tet repressor [74]. Plasmids were linearized, transfected into the parasites by electroporation, and permanent cloned cell lines were obtained by limiting dilution [75].

### Western blotting

Cells (5×10^6^ to 2×10^7^) were harvested by centrifugation and the pellets boiled in reducing SDS sample buffer before separation by SDS-PAGE. After Western blotting, tags were detected with the following primary antibodies at 1∶2000 dilution∶ mouse M2 (anti-Flag, Sigma), mouse anti-V5 (Invitrogen), mouse anti-myc (Santa Cruz Laboratories) and, for the TAP tag, peroxidase anti-peroxidase (PAP, Sigma). Detection was done using the ECL kit (GE Healthcare). In order to visualise *Tb*UPF1 in trypanosomes, we raised antibody against N-terminally truncated *Tb*UPF1 (residues 396–842), but the serum failed to detect the protein on Western blots.

### Immunofluorescence staining

Approximately 10^6^ cells were collected by centrifugation and resuspended in 50 µl of PBS. Cells were fixed in 4% paraformaldehyde in PBS for 20 min, washed twice in PBS and allowed to settle onto poly-lysine-coated slides. Cells were permeabilised in 0.2% Triton-X100 in PBS, washed twice and blocked in 0.5% gelatine in PBS for 1 hour. Primary antibody was added at the manufacturer-recommended dilution, incubated for 1 hour and the slides washed 3 times in PBS before addition of Alexa -594-conjugated secondary antibody (1 hour). Slides were washed, stained with DAPI (10 min), and washed twice more before drying the slide and mounting with Vectorshield.

### Northern blotting and RNA half-life assay

Parasites were harvested at room temperature and RNA isolated by Trizol extraction. RNA was denatured with formamide and formaldehyde, separated on a denaturing formaldehyde gel, blotted onto Nytran and hybridized. The DNA probes were made from plasmids or PCR products, by random priming with ^32^P label dCTP.

For RNA half-life assays, synthesis and maturation of mRNA were inhibited by addition to the growth medium of 2.0 µg/mL Sinefungin then, 5 min later, 10 µg/mL Actinomycin D [59], [76]. The time of Actinomycin D addition to the cell was taken as time 0. Cells were collected at various times and RNA isolated by Trizol extraction. RNA levels were estimated by Northern blotting using [^32^P]-labelled probes, and quantitated by phosphorimaging. The *7SL*RNA, or mRNAs encoding histone H4 (*HISH4*) or beta-tubulin (*TUB*) were used as loading controls. To obtain half-lives, we used the phosphorimaging results starting 2 h after Actinomycin D addition. Half-lives were measured for each individual experiment and, if there were at least three experiments, expressed as arithmetic mean ± standard deviation. For two experiments an average is given.

### Transcriptome analysis

Microarray analyses to compare the RNA of wild-type and *TbUPF1*-depleted procyclic forms (cell line with RNAi targeting the entire *TbUPF1* ORF) were done using 6 slides, with two biological replicates, as described in [77]. Almost no differences were found, so the results have not been uploaded into a public database.

### Real-time PCR

Real time PCR was performed using the SYBR green I kit (Roche Applied Science). RNA was isolated using Trizol. About 1 µg total RNA was DNase treated, then cDNA was synthesised using SuperScript III (Invitrogen). After RNaseH digestion of the DNA-RNA hybrid, the cDNA was diluted and used for real time PCR in the LightCycler^R^ 480 system. Standard curves for each primer set - CZ 3634 and CZ 3633 for *CAT* and CZ 3636 and CZ 3635 for the reference neomycin phosphotransferase (*NEO*) gene were included during each run to calculate the in-run PCR efficiency. Primers were designed using Primer3 software [78]. Relative quantification was done according to the E (efficiency) method using the LightCycler^R^ 480 software (Roche Applied Science).

### Co-immunoprecipitation

Procyclic cell lines carrying V5-tagged *Tb*UPF1, either alone or with co-expressed *Tb*UPF2-myc, were induced with 100 ng/ml tetracycline for 48 hours in medium lacking selective antibiotics. For each sample, 6×10^8^ cells were harvested and suspended in 2 ml of lysis buffer containing 20 mM Tris-HCl pH 7.4, 10 mM NaCl, 0.1% IGEPAL, 100 nM okadaic acid and Complete protease Inhibitor without EDTA (Roche). The cells were lysed by passing through a 21-gauge syringe several times on ice. Cell lysis was monitored under the microscope. The salt concentration was adjusted to 150 mM NaCl, and the lysate clarified by centrifugation at 13,000 rpm, 20 min at 4°C (microfuge). The cleared lysates (1 ml each) were treated with to RNase A (200 µg/mL) or 4 mM Vanadyl Ribonucleoside complexes (VRCs, Sigma). About 800 µL of each lysate (± RNase A) was incubated with 50 µL of Myc-agarose (Sigma) for 2 h at 4°C with rotation. Immune complexes were washed 3 times with 500 µL of 20 mM Tris-HCl (pH 7.4), 150 mM NaCl, 0.1% IGEPAL, and 100 nM okadaic acid, boiled in 80 µL of 2× SDS sample buffer, then analyzed by Western blotting. For the input and flow-through fractions, 2.5% of the total (equivalent to 6×10^6^ cells) was loaded. The Western blot was probed with anti-V5, anti-myc or anti-PABP antibodies (gift from Dr. Laurie Read).

## Supporting Information

Figure S1 **Sequence alignment of** ***Tb*** **UPF1 with UPF1s from other species.** Residues that are100% conserved are in solid red boxes, while those in which at least 70% are similar are outlined in red. The arginine residue at position 747 in the trypanosome sequence was mutated to make upf1-1. Other arginines in this region that were shown to be essential in yeast or human UPF1 are indicated by asterisks. The Figure was generated by Clustalw2 and ESPript. Sequences used were: Tb: *Trypanosoma brucei* CP000068.1. Sc: *Saccharomyces cerevisiae* NC_001145.2. At: *Arabidopsis thaliana* AF484122.1. Tv: *Trichomonas vaginalis* DS113229.1. Dd: *Dictyostelium discoideum* XM_631398.1. Gi: *Giardia intestinalis* DQ861427.1. Tg: *Toxoplasma gondii* XM_002368483.1. Pf: *Plasmodium falciparum* AE014185.2. Hs: *Homo sapiens* NM_002911.3.
(PDF)

Figure S2 **The strategy for replacement of the** ***GPI-PLC*** **gene with a mutant copy.** In wild-type cells the *GPI-PLC* gene (pink) is flanked by *HSP100* (green) and *ß′-COP* genes. The poly(A) site is marked as “a” and the two alternative spliced learder addition sites as black dots (“*SL*” for “spliced leader”). The panel below shows the loci in the double knockout line, and below that the construct that was used to return the full-length or PTC versions of *GPI-PLC*. The bottom panel shows the loci in cells containing the integrated return construct.
(PDF)

Figure S3 **RNA degradation kinetics for selected** ***CAT*** **constructs, including the effects of** ***UPF1*** **RNAi,** ***UPF2*** **RNAi, or tethering of PABP.** Results are shown as arithmetic mean ± standard deviation for 3 or more experiments, with all values at +2 h set as 100%. When two experiments were done, each is shown individually. The curves were fitted for time points from +2 h onwards. The half-lives shown were calculated separately for each individual experiment and – if there were three experiments - are presented as arithmetic mean ± standard deviation. The constructs used are indicated above the graphs; the presence of a tethered protein or induction of RNAi are also indicated.
(PDF)

Figure S4 **Tethering of UPF1 to T7-transcribed** ***CAT-B-ACT*** **mRNA increases the mRNA degradation rate.** The Figure show the results for two inependent experiments, done using the clones shown in Figure 5C, and as described for Figure S4. Expression of lambdaN-UPF1-flag was induced with tetracycline. In the absence of lambdaN-UPF1-FLAG induction, the half-life of the *CAT-B-ACT* mRNA was 15–20 min. This was surprisingly short. We do not understand why, but one possible explanation is that the mRNAs were made by T7 polymerase. Instability of a T7-produced *CAT* reporter with a different 3′-UTR has been previously observed; the reasons are unknown (Colasante et al., Mol Biochem Parasitol 151, 193–204.) Upon induction of lambdaN-UPF1-FLAG expression the half-life decreased further.
(PDF)

Figure S5 **RNAi targeting** ***TbUPF1*** **has minor effects on the abundances of mRNAs from two loci.** (A) shows a map of the region around Tb927.10.12900. The direction of transcription and the positions of various probes (a–d, dotted lines) used to identify the various RNAs is indicated. Estimated sizes and location of monocistronic RNAs, based on our own mapping and on RNASeq tag abundances shown at tritrypDB, are also shown. (B) Shows a Northern blot of RNA with and without RNAi targeting either *XRNA* or *UPF1*, using probe (a) (made using CZ3585, CZ3586). A Histone H4 probe (*HISH4*) serves as a control. Probe ‘c’ (made using oligonucleotides CZ3695, CZ3696) detected a single RNA migrating at 3.7 kb and probe ‘d’ (made using CZ3707, CZ3708) detected two RNAs of similar abundance, migrating at 2.6 kb and 1.8 kb. The band of 4.5 kb was detected only by probes (a) and (b). There is a tag gap in the middle of Tb10.389.0620, but no mapped spliced leader addition site. (C) The procyclin locus containing *EP1* and *EP2*, with predicted transcripts based on mapped splicing sites. Alternative splicing of the procyclin *EP2* RNA precursor is known to result in two transcripts. The dominant one, at 0.9 kb, has a short 5′-UTR preceding the *EP2* initiation codon. The minor one, of 1.4 kb, has various very short ORFs upstream of the *EP2* ORF while the 0.4 kb RNA contains these short ORFs alone: either of these might be a substrate for NMD. The location of the *EP2* upstream probe used in (D) is indicated as a dotted line. (D) RNA was prepared from three independent bloodstream-form trypanosome clones (AnTat1.1. strain) with *TbUPF1* hpRNAi, incubated with 100 ng/ml tetracycline for 2 days. The blot was probed with the *EP2* upstream region indicated in (A). We detected the 0.9 kb *EP* mRNAs and three additional bands at 0.4, 1.4 and 2.3 kb. The 2.3 kb signal comigrates with rRNA so could either be a cross-hybridisation or a dicistronic precursor. The bands were all of approximately equal intensity: the mature *EP* mRNA is very unstable in bloodstream forms. The level of the 1.4 kb *EP* mRNA increased 1.3–1.4 fold after *TbUPF1* depletion, whereas the levels of the monocistronic 0.9 kb mRNA and the 0.4 kb RNA were unaffected. The *uORF-EP2* RNA probe was made by in vitro transcription using ^32^P-UTP and T7 RNA polymerase, from a PCR template carrying the T7 polymerase promoter sequence. (The primers were CZ3391 and CZ3392.).
(PDF)

Table S1 **Plasmids used in this study.**
(DOC)

Table S2 **Oligonucleotides used in this study.**
(DOC)
