# Supplementary material for: Is There a Classical Nonsense-Mediated Decay Pathway in Trypanosomes?
Source: PLoS One. 2011 Sep 21;6(9):e25112. doi: 10.1371/journal.pone.0025112 (PMC3177853; doi:10.1371/journal.pone.0025112)
Supplement: Figure S1 — Sequence alignment of Tb UPF1 with UPF1s from other species. Residues that are100% conserved are in solid red boxes, while those in which at least 70% are similar are outlined in red. The arginine residue at position 747 in the trypanosome sequence was mutated to make upf1-1. Other arginines in this region that were shown to be essential in yeast or human UPF1 are indicated by asterisks. The Figure was generated by Clustalw2 and ESPript. Sequences used were: Tb: Trypanosoma brucei CP000068.1. Sc: Saccharomyces cerevisiae NC_001145.2. At: Arabidopsis thaliana AF484122.1. Tv: Trichomonas vaginalis DS113229.1. Dd: Dictyostelium discoideum XM_631398.1. Gi: Giardia intestinalis DQ861427.1. Tg: Toxoplasma gondii XM_002368483.1. Pf: Plasmodium falciparum AE014185.2. Hs: Homo sapiens NM_002911.3. (PDF) [file pone.0025112.s001.pdf]

Supplementary Figure 1

Sequence alignment of TbUPF1 with UPF1s from other species. Residues that are 100% conserved are in solid red boxes, while those in which at least 70% are similar are outlined in red. The arginine residue at position 747 in the trypanosome sequence was mutated to make upf1-1. Other arginines in this region that were shown to be essential in yeast or human UPF1 are indicated by asterisks.

The Figure was generated by Clustalw2 and ESPript. Sequences used were:

Tb: *Trypanosoma brucei* CP000068.1

Sc: *Saccharomyces cerevisiae* NC_001145.2

At: *Arabidopsis thaliana* AF484122.1

Tv: *Trichomonas vaginalis* DS113229.1

Dd: *Dictyostelium discoideum* XM_631398.1

Gi: *Giardia intestinalis* DQ861427.1

Tg: *Toxoplasma gondii* XM_002368483.1

Pf: *Plasmodium falciparum* AE014185.2

Hs: *Homo sapiens* NM_002911.3

```
TbUPF1        ............................................................
ScUPF1        ............................................................
HsUPF1     1  ..................MSVEAYGPS.....SQTLTFLDTEEAELLGADTQG.....SE
AtUPF1     1  ...............MDSQQSDLFDTA.....SQPDTVAD..EYTFLEFNTQGD....SE
TvUPF1        ............................................................
DdUPF1     1  ............MYGFNIGKAENAGLHLDNKKSENNNSSNNELHKVINTINNGFQFKDDD
GiUPF1     1  MEPCALCGSLAEPLVRCGACREVYCNNFDHKTSHIVRHLQLTGHDHIEPVSEFYIPSDVL
TgUPF1     1  ..........MASPNSFDISDFIFDAADLNFTPNGGAVSTVPGVDVDEDFSSPLPPSGPT
PfUPF1     1  .........MDKTFDYNNIHVDNFDELYDFCISKDTNKKKIKEENYFSEDTQQKFNDDKD

TbUPF1        ............................................................
ScUPF1     1  .......................................MVGS..............GSH
HsUPF1    33  FEFTDFTLPSQTQTPPG..........GPGGPGGGGAGGPGGA..............GAG
AtUPF1    35  FDYQDFGSPTAWPTPSDSISIADVADRGEGGAAADHHSEASSP..............SSL
TvUPF1     1  .........................................................MTE
DdUPF1    49  DEDNENNLNNESSSDEENQQQQNNNNNNNKNQFEDDDEDQEEEEYISGDDLNNNGQESDD
GiUPF1    61  YCNDCETKGITSLGFLPTKADACSGIICCVNCRMYHDQTHDTSAWRPIISTHIDSRFFPG
TgUPF1    51  ARLDSYPPSSGRNTSDEAYQQGDASTASPQRCPLPSGGVSGDGAVEDLQEKLDRLHLAPE
PfUPF1    52  TCNKEVLKKNTNIKPTKKHGKNKKSEYSPCKIIEEKRLDMLNESLEDTESYYDRKKKNSH

TbUPF1        ............................................................
ScUPF1     8  TPYDISNSPSDVN...............................................
HsUPF1    69  AAAGQLDAQVGPEG..............................................
AtUPF1    81  SAGAGNGAKVGRGG..............................................
TvUPF1     4  KKQANDRAWSANDN..............................................
DdUPF1   109  YEEEEEDDDEDEEEEDEESKKLNYDSFFG...............................
GiUPF1   121  HEEAENQGDEPNVGDCTFTGVESYYTPLP...............................
TgUPF1   111  EDWSNLHSGPGPSG..............................................
PfUPF1   112  KYKEINNSNNNNNNNNNNNDDNNSKKHIDPHGNDCIHQGKHKNEHEQQYYSNKHMKNNSE

TbUPF1     1  ..........MFSEHASDERALG...............VATTP..VASGETNK.......
ScUPF1    21  ....VQPATQLNST.LVED............................DDVDNQLFEEAQV
HsUPF1    83  ....ILQNGAVDDS..VAKTS..........................QLLAELNFEE.DE
AtUPF1    95  ....VGGSGGVSSSSQVDALA..........................AGVGNLNFEE.TG
TvUPF1    18  .....AVKGSVGYTPKED.............................PTIDDEPQEE...
DdUPF1   138  ..ANMSHNMDFLNSQNINSQIG....................LMDPHLHSSQLNFEE...
GiUPF1   150  ....STEMTSKDSIVPSVHDILNDDLLFTMDTYRSNLFSSLSPMEAQPHERQTFLEMIED
TgUPF1   125  ...SVDASVATLSSLAERQETVGRSGSGEVPESHRKCAEDGTPNGVHAGDAGEPREELKE
PfUPF1   172  CLSDISSYTDIRSCNNVEDGIYDEYDLTNKMKYEKTNKYKNKNKNKMDDTHNELYEEGNN

TbUPF1    27  .............KPSCSYCSEESPTCLAFCNGCS.......KWFCN....GSNGTSGSH
ScUPF1    48  TETGFRSP..SASDNSCAYCGIDSAKCVIKCNSC.......KKWFCN....TKNGTSSSH
HsUPF1   110  ED...TYYTKDLPIHACSYCGIHDPACVVYCNTS.......KKWFCN....GRGNTSGSH
AtUPF1   124  DDDGFDYGKNDFTEHACKYCGISNPACVVRCNVASC.....RKWFCN....SRGNTSGSH
TvUPF1    41  .........LVIDRPCCSYCLCDIPECLVKDKAIG.......RWFCN....GRGKALHSH
DdUPF1   173  .....PQEEIELPAHACAYCATHELSTVVKCMHPSC.....GKWFCN....GKGKTKSSH
GiUPF1   206  RAASKVNDHLKLVDEDVPVVTDTALERLTKQSDATRNRRRIIKKSCIDDETARQEILASI
TgUPF1   182  DHGRGGKATKEIPEHACSYCGASSPDCVLKCCCCN.......KYFCNSPCSASGSSMGSH
PfUPF1   232  YRYKNKKSKDDLKYYRCRYCEIDSIDSVVQCNNC.......KRWFCN....GSYGTCGSH

TbUPF1    63  IILHLVKSGHNSLKLHAENSLGDSTLECYICRSSNIFSLGFMPSKEEAVVVLVCREPCLH
ScUPF1    95  IVNHLVLSHHNVVSLHPDSDLGDTVLECYNCGRKNVFLLGFVSAKSEAVVVLLCRIPCAQ
HsUPF1   156  IVNHLVRAKCKEVTLHKDGPLGETVLECYNCGCRNVFLLGFIPAKADSVVVLLCRQPCAS
AtUPF1   175  IVNHLVRAKHKEVCLHRDSPLGETILECYNCGCRNVFLLGFISAKTDSVVVLLCRDPCLN
TvUPF1    81  IIHHLVKSRHMEIELPPQNPYSQIPMTCYLCHSTNIFRLSFVQSQKTGKYFVLCRECCND
DdUPF1   219  IITHLVKSKHKEVALHPESSFGDTTLECFNCGCKNIFLLGFITARTESVVVLLCRDPCAS
GiUPF1   266  VLAIPYNMEPQTLEAVTARGCPAMVMWAIIDGLLPGFDFAIPRDKSTHVRTFVSKTDRCH
TgUPF1   235  IIFHLVKSRHREVMLHPEGPLGDCTLECFQCGSRNVFLLGLIPAEQEGVVVLICREPCLS
PfUPF1   281  IVTHLVRSKHKEIRLHKNSLLGETILECYNCACRNVFLLGFLPTSEEGVVVIICRDPCLS
```

```
TbUPF1   123  S...........................................................
ScUPF1   155  T...........................................................
HsUPF1   216  Q...........................................................
AtUPF1   235  V...........................................................
TvUPF1   141  P...........................................................
DdUPF1   279  G...........................................................
GiUPF1   326  ILSHFYCRRKLHYFHYMLPILALERLITRDQLEKNQVPVKGVQFRCLRGRWSVNNFDKSG
TgUPF1   295  S...........................................................
PfUPF1   341  SYISLYEKHVGPFDEHKSKSLQDDMEKEILLEDHDVDNEEINDEPNKNNKKKCENINSSN

TbUPF1        ............................................................
ScUPF1        ............................................................
HsUPF1        ............................................................
AtUPF1        ............................................................
TvUPF1        ............................................................
DdUPF1        ............................................................
GiUPF1   386  LATICGAMWEVLIPIQDISMDIKIYKGENFILRIIDYQGENPKGFTLYNG..........
TgUPF1        ............................................................
PfUPF1   401  EDEKEHEEPINFCSLNHPSDSSDYYPYYYYNHHSNYSRREKNKKHNKGSEKKHMNVCCDD

TbUPF1   124  ........................................................KTLR
ScUPF1   156  ...........................................................K
HsUPF1   217  ........................................................SSLK
AtUPF1   236  ........................................................NALK
TvUPF1   142  .........................................................QLH
DdUPF1   280  .........................................................PSK
GiUPF1   436  ...........................ILEIPLLCTSVNGDNVAFFVHQNSELQTSMPYN
TgUPF1   296  ........................................................GALK
PfUPF1   461  NKMKDCQNNNNPNKYVDDENMIDGKDNKKYEKKGKQNDVYKEDEKGSYINNSNINDELRK

TbUPF1   128  DLNWDSSTWLPLIE..................ERRLLPWICSIPSTLRRP.......LTL
ScUPF1   157  NANWDTDQWQPLIE..................DRQLLSWVAEQPTEEEK...LKARLITP
HsUPF1   221  DINWDSSQWQPLIQ..................DRCFLSWLVKIPSEQEQ...LRARQITA
AtUPF1   240  DMNWDLSQWCPLID..................DRCFLPWLVKVPSEQEQ...LRARQISA
TvUPF1   145  LYSLDLAHRQLIVQ..................QTQMLEWLVRPPSHAESKDGFRFCDITP
DdUPF1   283  EVNWDMSSWQPLINGG................EKAFCSWLVKTPSQVDS...ERSRQITI
GiUPF1   469  CVNYELMVKEDVVEPLTSEMAIDLKFTDIASPASYKHSHFISKPNLQYEIVKCDNDATYK
TgUPF1   300  QSGWDLTQWQPLIE..................GKSFLPWLVRSTLTAEEQ..RDCHVVTT
PfUPF1   521  IKDWDLKKWQPVIE..................DRFFLEWLVNIPSNEEAE..RKGKLSTS

TbUPF1   163  HDIKALEMSWEQKV.......................KEFVDPVES.......VPEVPLY
ScUPF1   196  SQISKLEAKWRSNK.......................DATINDIDAPEEQEA.IPPLLLR
HsUPF1   260  QQINKLEELWKENP.......................SATLEDLEKPGVDEE.PQHVLLR
AtUPF1   279  QQINKIEELWKTNP.......................DATLEDLEKPGVDDE.PQPVQPK
TvUPF1   187  TDMDLLEETWPKNP.......................NATILDLPQIRKSTT.IPKTKPT
DdUPF1   324  QQILRLEEFWKMDP.......................EATLLDIEAPRSDDEKPASTQLA
GiUPF1   529  RHVSAIARLCAYNPSVWSYLTSKKWTRVLNLSSEDFVDSSSERVRLTSFDCFIRFILLAD
TgUPF1   340  QQLQRLEELWQKNP.......................QATLEELSQTKEEAP.LPCVKLV
PfUPF1   561  YNVNKLEELWKNKK.......................DVYIDELNFEILNDE.PNKVELK

TbUPF1   193  FESGTKYVEVFSS...........LIALDSQGARDSKD.TSFEGIQCTQQKKIGGR..HF
ScUPF1   232  YQDAYEYQRSYGP...........LIKLEADYDKQLKESQALEHISVSWSLALNNRHLAS
HsUPF1   296  YEDAYQYQNIFGP...........LVKLEADYDKKLKESQTQDNITVRWDLGLNKKRIAY
AtUPF1   315  YEDAYQYQNVFAP...........LIKLEADYDKMMKESQSKENLTVRWDIGLNKKRVAY
TvUPF1   223  YKDIRDYATTYNT...........LVKLEMDYDKQVTESMIYRNVKINFKREGYNRFTGT
DdUPF1   361  YKDAYEYREIISP...........LIELEAKHEKELRESLSQSGISIEWSQGINKRYTAT
GiUPF1   589  YRMAHAFMDLYKQGVVTRYGEVLDGIKTRTTDRKAVKDVIPQDKYSANSDPLIVHKFFNL
TgUPF1   376  YEDGFDYQRTFAP...........LVQAEADFDKQIKDGQKLVRVKLRWEQGLNRRRLAY
PfUPF1   597  YEDAHHYQSIFSP...........LVQLEADYDKSIKEGQKQGNVSVRWDIGLNKKRYAH
```

```
TbUPF1   239  FVLKPFPLFDVGVNR..GDNVSIRVKGSESSLSGTITEVSATS.................
ScUPF1   281  FTLSTFESNELKVAI..GDEMILWYSGM................................
HsUPF1   345  FTLPKTDS.DMRLMQ..GDEICLRYKGD................................
AtUPF1   364  FVFPKEEN.ELRLVP..GDELRLRYSGDA...............................
TvUPF1   272  FSFPISET.SRPINI..GDTFLVKCGAY................................
DdUPF1   410  FPFSRSDL.EFKVVP..GDELKLQFISSTG..............................
GiUPF1   649  EEQFKQLNMDVTAELDRNEQMLIESRGNSRSTKGLQSSSGSSS.................
TgUPF1   425  FMYSRDEGCNVRVAA..GDEVKISTVLPKSVLSGASSSAPASSGGSHGSGCTYSNEATSS
PfUPF1   646  FIYIKEES.ELRLVA..GDELKISYTYP................................

TbUPF1   280  ..VDN.......EHAVFVTDTTARSVDKKAVN.EILAATTVTISPEYNGVAD........
ScUPF1   307  ...............QHPDWEGRGYIVRLPNSFQDTFTLELKPSKT...PPPTHLTTGFT
HsUPF1   370  ...............LAPLWKGIGHVIKVPDNYGDEIAIELRSSVG....APVEVTHNFQ
AtUPF1   390  ...............VHPSWQSVGHVIKLTA..QEEVALELRANQG....VPIDVNHGFS
TvUPF1   297  ....................EAKGSLERTLGVGEIELLFIRQPTPP........EDAIFT
DdUPF1   437  ...............GVIEWEDTGRVIHIDD..ENLLSLETKSRCS....FDSGPKGSYR
GiUPF1   692  ...............FCREGIDAFQILSFAATNQKKKLSTLPWNTPYKAMEEDNLDPGLI
TgUPF1   483  NGVGSGGTAGLEGDGNFVQWSCTGSITRFSED.SEEVIVEVKKPPNAKGAWDSPVPLLYT
PfUPF1   671  ...............DGSVWCCEGHISRLHN..TEEISLELRTSCTSNGPWVDNITTGFT

TbUPF1   322  ............KRKMEALQQFARS.EGSVSAYLYFTILGQ.KERAAHRN...SGFD...
ScUPF1   349  AEFI..WKGTSYDRMQDALKKFAID.KKSISGYLYYKILGH.QVVDISFD...VPLP...
HsUPF1   411  VDFV..WKSTSFDRMQSALKTFAVD.ETSVSGYIYHKLLGH.EVEDVIIK...CQLP...
AtUPF1   429  VDFV..WKSTSFDRMQGAMKNFAVD.ETSVSGYIYHQLLGH.EVEAQMVR...NTLP...
TvUPF1   329  VQLV..WLDTSFVRMIGAIAKMPQSPQTSTANIIKEVIMGHLPDTIPTLPGEPN......
DdUPF1   476  MEMV..WRSTSSERILSAMKSFAIK.EQALSSYLYHALLGHPDIPPAPLD...IQLP...
GiUPF1   737  EKYANNFAHLTKGVKVRSSEDFDINEVLKRASCLMPKTLRSKADTERLISELSAFLD...
TgUPF1   542  IEFV..WKSTSFERMQAALRQLAVD.EISVSSYLYHTLMGK.QMEHQIIQ...TPMP...
PfUPF1   714  VEFI..WKSTAYDRMQLALNEFALN.SYSLSGFLYHKLLGH.DISEDSLE...YNKNTFH

TbUPF1   362  ..........TEPEPRGHHNLNYSQEQALRVALRNPLTLIQGPPGTGKTSTS......VA
ScUPF1   399  ..........KEFSIPNFAQLNSSQSNAVSHVLQRPLSLIQGPPGTGKTVTSATIVYHLS
HsUPF1   461  ..........KRFTAQGLPDLNHSQVYAVKTVLQRPLSLIQGPPGTGKTVTSATIVYHLA
AtUPF1   479  ..........RRFGVPGLPELNASQVNAVKSVLQKPISLIQGPPGTGKTVTSAAIVYHMA
TvUPF1   381  ..........RSPVVKGIPTLNLSQVNAVSYALKSPFCMIQGPPGTGKTTTIAALVTRFL
DdUPF1   527  ..........TNFHLKNLPRLNESQISAVNKVLTAPLSLIQGPPGTGKTVISSFIIHHLV
GiUPF1   794  .............TLEEKVTLNFSQKDVIQYVLSRPITLVQGPPGCGKTFIGACLAWLFS
TgUPF1   592  ..........LQISAPNLAPLNPSQMLAIRYALQHPLSLIQGPPGTGKTLTCSTLVYQMV
PfUPF1   767  KLMHKKVMSIRNYSAPNLAPLNHSQIDAIKRSLLSPLSLIQGPPGTGKTLTCATLVYHLV

TbUPF1   406  IIRELHSHVKSR...........ILVCAPSNVAVDHLAQRVSGTGLKVVRLQAKYRNDIP
ScUPF1   449  KIHKD.................RILVCAPSNVAVDHLAAKLRDLGLKVVRLTAKSREDVE
HsUPF1   511  RQGNG.................PVLVCAPSNIAVDQLTEKIHQTGLKVVRLCAKSREAID
AtUPF1   529  KQGQG.................QVLVCAPSNVAVDQLAEKISATGLKVVRLCAKSREAVS
TvUPF1   431  QAKAG.................PVLVCAPSNAAVERVTEAIASTHASVCRVISTSRTDIE
DdUPF1   577  KYVKGN...............DKVLVCTPSNVAIDQLTGKLHEIGLKVVRLSSKLREEVA
GiUPF1   841  KVYGSLDHDSGS...FRQATPVPVLICCPSNTAAESLTLALEPFGLPVVRVVSLARQRLA
TgUPF1   642  KLSEVGSHIHPRCAGRVNKEGGQVLVVAPSNVAVDQLAERINRTGLKVIRMYSKSREGAS
PfUPF1   827  KMNMG.................KVLVTAPSNVAVDQLSVRIHRSGLKVVRLCSKSRESVP

TbUPF1   455  CS..........VESIGLERQVGDYINASSGLERLKE.......................
ScUPF1   492  SS..........VSNLALHN.....LVGRGAKGELKN.......................
HsUPF1   554  SP..........VSFLALHNQI..RNMDS..MPELQK.......................
AtUPF1   572  SP..........VEYLTLHYQV..RHLDTSEKSELHK.......................
TvUPF1   474  AID..........DKYALHNMV..YSLDCAESRRLND.......................
DdUPF1   622  SP..........VEHLTLHKQV..YKLDQMGDGELGK.......................
GiUPF1   898  EYEESPNTYADRVCLHVLFEEILKLTLGDDAPSDFTPSKEARLIYENMLDEIPVKQFNEV
TgUPF1   702  SSLTSF.....CVENLALHKKVLELKTGSS..DEMAK.......................
PfUPF1   870  SI..........AEYLYLHNQM..KLLKTDIAEELNK.......................
```

```
TbUPF1    482  .....LLDSMQTGKSLNDKDYGTYKDGVEKIERLILRNADVVCCTCIGAGDYRLKTMKFK
ScUPF1    514  .....LLKLKDEVGELSASDTKRFVKLVRKTEAEILNKADVVCCTCVGAGDKRLDT.KFR
HsUPF1    577  .....LQQLKDETGELSSADEKRYRALKRTAERELLMNADVICCTCVGAGDPRLAKMQFR
AtUPF1    597  .....LQQLKDEQGELSSSDEKKYKNLKRATEREITQSADVICCTCVGAADLRLSNFRFR
TvUPF1    499  .....MLIERSN.RDFSEDEEKKFKDLRKSLENRVIDAADVITCTCITSADPRLATKVFP
DdUPF1    647  .....LRKLKEAFGSLSNEDEKRYIYLRRMMEMAILRKADVICATCVGAGDPRLSQFRFP
GiUPF1    958  YNYLQMKKKENIDVALKQSAEEEISKAMFEIENIIISSAKVVVCTCSTSYDNHLSRVHFS
TgUPF1    732  .....YIQLKEQTGELAAADERRLRLLISRAEMEILQTADVICTTCVGAGDNRLQGFRFR
PfUPF1    895  .....LLELKEEVGELSQKDERRLKKLILFAEHEILIEADVICCTCVGAMDKRLKKFRFR

TbUPF1    537  HVLIDEATQGTEPEVLIPLVRGAKQVILVGDHCQLRPLVFSTAAEKAGYQRSLFERLVLM
ScUPF1    568  TVLIDESTQASEPECLIPIVKGAKQVILVGDHQQLGPVILERKAADAGLKQSLFERLISL
HsUPF1    632  SILIDESTQATEPECMVPVVLGAKQLILVGDHCQLGPVVMCKKAAKAGLSQSLFERLVVL
AtUPF1    652  QVLIDESTQATEPECLIPLVLGVKQVVLVGDHCQLGPVIMCKKAARAGLAQSLFERLVTL
TvUPF1    553  TVIIDEATQAVEPEILIPIMHGSKQVCLVGDHMQLGPVVTNPKCVEAGLGNSIVQRLVQL
DdUPF1    702  HILIDESTQASEPECLIPLMMGAKQVILVGDHRQLGPVLLCKKVVDAGLSQSLFERLISL
GiUPF1   1018  SLIVDESTQAIEPDTLCAIGHGCSHIVLMGDHKQLGPIVATNIARHSKLDLSLYERLQRA
TgUPF1    787  QVVIDEAAQATEPECLIPIVLGAKQVVLIGDHCQLGPVVLSKKAAAAGLATSLFSRLLAL
PfUPF1    950  QVLVDEATQSTEPECLVPLVTGAKQIVLVGDHCQLGPIIVCKKAANAGLGKSLFERLVML

TbUPF1    597  GHRPVRLDVQYRMNPSLSFFPSHHYYEGTLQNGVTAEQR......DASEVFPWPDVTKPI
ScUPF1    628  GHVPIRLEVQYRMNPYLSEFPSNMFYEGSLQNGVTIEQR.....TVPNSKFPWPIRGIPM
HsUPF1    692  GIRPIRLQVQYRMHPALSAFPSNIFYEGSLQNGVTAADR.....VKKGFDFQWPQPDKPM
AtUPF1    712  GIKPIRLQVQYRMHPALSEFPSNSFYEGTLQNGVTIIER.....QTTGIDFPWPVPNRPM
TvUPF1    613  GLRPQRLLTQYRMHPVLSEFPSNTFYDGELMNGIPAEKR.....TPQQPVFNWPKPSFPL
DdUPF1    762  GHHPERLTIQYRMHPSLTEFPSNTSYEGQLVSELSHTDR.....DSQ.SKFPWPQPKDPM
GiUPF1   1078  GIEPHSLTVQYRMHPALSAFPSNTFYNGMLQNGVTQADRQLIPKPLSIDSFPWPIPSTPS
TgUPF1    847  GHRPLRLKVQYRMHPALSFFPSYFFYEGELQNGVTMTERTYFHQGPGDHRFPWPNEERPM
PfUPF1   1010  GITPFRLEVQYRMHPALSEFPSYVFYDGSLQNGITLKER.....EYPLKSFPWPNAKCPM

TbUPF1    651  FFYNATGNEE.LGSNGRSYLNRAEAALTEQIVTKLIQG.GVEPGDIGVITPYRSQCRYLR
ScUPF1    683  MFWANYGREE.ISANGTSFLNRIEAMNCERIITKLFRD.GVKPEQIGVITPYEGQRAYIL
HsUPF1    747  FFYVTQGQEE.IASSGTSYLNRTEAANVEKITTKLLKA.GAKPDQIGIITPYEGQRSYLV
AtUPF1    767  FFYVQLGQEE.ISASGTSYLNRTEAANVEKLVTAFLKS.GVVPSQIGVITPYEGQRAYIV
TvUPF1    668  MFYNNVNNEEEISNSGTSYINAFEATIVSQIVTQLCKA.GVDPQQIGIISPYSGQKFYLQ
DdUPF1    816  FFFNCTGSEE.ISSSGTSFINTTEASICEKIVTKFLEL.GSLPGQIGIITPYEGQRAYIT
GiUPF1   1138  FFWHVQGTHE..IGHGTSLRNDTEILCVEAIVDQLLKCYELKQSDIGIVTPYDYQKCQIE
TgUPF1    907  FFYHSTASEE.ISGSGTSYVNRIEASNIEKIVTFLLKC.GLKASQIGVITPYDGQRAHIS
PfUPF1   1065  FFYNSTGLEE.MSASGTSYLNRSEASNMEKLVRTLLQC.GLKPSQIGVITPYEGQRAYIT

TbUPF1    709  SYLSRSGRLPMEVYDRVEISSVDAFQGREKEFIILSCVRSNHRQGAGFVTDGRRLNVSLT
ScUPF1    741  QYMQMNGSLDKDLYIKVEVASVDAFQGREKDYIILSCVRANEQQAIGFLRDPRRLNVGLT
HsUPF1    805  QYMQFSGSLHTKLYQEVEIASVDAFQGREKDFIILSCVRANEHQGIGFLNDPRRLNVALT
AtUPF1    825  NYMARNGSLRQQLYKEIEVASVDSFQGREKDYIILSCVRSNEHQGIGFLNDPRRLNVALT
TvUPF1    727  NFLASMATLPSSFYQRLAIASVDSFQGGEKDYIIMSCVRCNPHGSIGFLKDYRRLNVALT
DdUPF1    874  SHMQKSGKLNLELYKSIEVASVDSFQGREKDYIILSCVRSNDYQGIGFLQDPRRLNVALT
GiUPF1   1196  MQLKDAG......YSEVFVNSVDAFQGHEKEVIIFSTVRSVDKH.IGFLKDQRRLNVGLT
TgUPF1    965  SLFQRQTTLGQAAFADLEVASVDAFQGREKDFILLSCVRSNSNTGIGFLADSRRLNVAMT
PfUPF1   1123  SLFQKNISFQNSS..DIEVASVDAFQGREKDFILLSCVRSNKKLGIGFLNDPRRLNVALT

TbUPF1    769  RAKRGLIIMGNVQLFSRYPG........................................
ScUPF1    801  RAKYGLVILGNPRSLAR...........................................
HsUPF1    865  RARYGVIIVGNPKALSK...........................................
AtUPF1    885  RARYGIVILGNPKVLSK...........................................
TvUPF1    787  RAKYGLIIVGCARVLSK...........................................
DdUPF1    934  RARFGLIILGNAKVLSK...........................................
GiUPF1   1249  RCRCALIIVGNATALAID..........................................
TgUPF1   1025  RAKYGLIICGNATVLANYMPRVARPPGAPAAPPPGGGTTGLPGNALGPCGVQVGPGASTL
PfUPF1   1181  RAKYGLIICGNAKVLSRHHFISREKINANET.............................
```

```
                            .         .         .         .
TbUPF1    789   ...........WHELLVHMNSLSLIVEGPIDDLVPSAVVLQKP.............RKRG
ScUPF1    818   ........NTLWNHLLIHFREKGCLVEGTLDNLQLCTVQLVRPQPRKTERPMNAQFNVES
HsUPF1    882   ........QPLWNHLLNYYKEQKVLVEGPLNNLRESLMQFSKPRKLVNTINPGARFMTTA
AtUPF1    902   ........QPLWNGLLTHYKEHECLVEGPLNNLKQSMVQFQKPRKIYNDRRLFYGGGAGM
TvUPF1    804   ........SILWYNLLRHCQEQHVLVEGSIMDLKESPCVLQKPSVKQSEIFPGIPVGEFG
DdUPF1    951   ........DPLWNSLISHFKNKNVLVEGSLANLKQSPVILQKPKKLYGQGKLPIPGQNSN
GiUPF1   1267   .........STWRSLIQHYYNNKVLVMGRDLDQLFQIESLDEVIPKA.............
TgUPF1   1085   SGTPCVPEPPIWRLLIHHYLKYDLVVDGPLSNLKPSKIRITLPPLTPFGALTAAGASKGG
PfUPF1   1212   ....ITNVNSVWINLLSQFKKKDLIVEGCLANLKPVNINIPTPTKLPSRYTNFNYFHSYD

                             .         .
TbUPF1    825   ........GKLKGEESDSPLFLPGES..................................
ScUPF1    870   EMGDFPKFQDFDAQSMVSFSGQIGDFGNAFVDNTELSS...YINN...............
HsUPF1    934   MYDAREAIIPGSVYDRSSQGRPSSMYFQTHDQIGMISAGPSHVAAMNIP.IPFN......
AtUPF1    954   IGNDNFGSGNPNADRRGSRGRAGGSYLPSGPPNGARPG..LHPAGYPIPRVPLSPFPG..
TvUPF1    856   KDMVPNTVESNFDNDDNDGEAYFNTVS.................................
DdUPF1   1003   SFNYDREHIDPNIGMNMVYGISNNNINNNSNNINNNNNNNINNNINNNNNQRFNTQDGRY
GiUPF1          ............................................................
TgUPF1   1145   RGGAGATKGKLGKIKAASRHIAPEELQDYDRHATTSGYLQTVPSSGGLSAGQPNAGPPPA
PfUPF1   1268   NFQKNKTYSYQDKFKSNTYKDNNTNTTNQYNNNNNNNN...NNNNNKNDDQLFNSSHN..

TbUPF1          ............................................................
ScUPF1    912   .........EYWNFENFKSAFSQKQNRN................................
HsUPF1    987   ..........LVMPPMPPPGYFGQANGP.....AAGRGTPKG.KTGRG.......GRQKN
AtUPF1   1010   ....GPPSQPYAIPTRGPVGAVPHAPQPGNHGFGAGRGTSVGGHLPHQ.......QATQH
TvUPF1          ............................................................
DdUPF1   1063   SNSQTSSSSQTYYGSTNSSGNTINSNQNQFFNTPSSYSANTGSSYQYRFQQYQQLQQPQP
GiUPF1          ............................................................
TgUPF1   1205   STYIPNGAGGYYSAPPLYPCAAFAALLGADRGFQSQSPMPSSSLMAAHAASGTAFYYPSG
PfUPF1   1323   ....NHYYSSYYYYSHFNKNHLPDENTNSQFNLNLSNYSYCSDHFMNNDLYNYINKNKKK

TbUPF1          ............................................................
ScUPF1    931   ...........EIDDRNLYQEEASHLN.........................SNFARELQ
HsUPF1   1024   RFGLPGPSQ...TNLPNSQAS.QDVASQP....................FSQGALTQGYI
AtUPF1   1059   NVGTIGPSLNFPLDSPNSQPSPGGPLSQPGYGSQAF..........RDGFSMGGISQDFL
TvUPF1          ............................................................
DdUPF1   1123   QQQQQPQQQQQPQQQQQPQQPQQQQPQQQKQYQQQQQQ.....QQQQQQQQQQQQQQQKQ
GiUPF1          ............................................................
TgUPF1   1265   QGGALAAAAVSGADHQTLRAPAAYFSGRDAEAGG............FSGGSQGASAACAS
PfUPF1   1379   NIVNSYVNNLSQMSQFNDYPTETNHNNDDHNYLHLHDKYNDTLDDDKKSSNKCNISSSLN

TbUPF1          ............................................................
ScUPF1    955   REEQKHELSKDFSNLGI...........................................
HsUPF1   1060   SMSQPSQMSQPGLSQPELSQDSYLG............................DEFKSQI
AtUPF1   1109   ADDIKSQGSHDPYNMADFATQASPGGFAVDYATQGAHGAFPGNFMNQNSQGGYSRFSGIN
TvUPF1          ............................................................
DdUPF1   1178   QPHQQYQSQKQQQQQQYQQPQQYQQQNQQYHQSQLPPKQYHNQRFNNNNNNNINNNNNNN
GiUPF1          ............................................................
TgUPF1   1313   SSFRDSGAGLGQSGAGASKSNTRPGEGPHGAVDVISRAIAGGGAFASRPFPTETADAGTA
PfUPF1   1439   NSEFSYPNNKMSSNHSIDSYNHDTNLISMSKNADNLLPYLFYYKNNQNSSSQPYMYYNQN

TbUPF1          ............................................................
ScUPF1          ............................................................
HsUPF1   1092   DVALSQDSTYQGERAYQHGGVTGLSQY.................................
AtUPF1   1169   DFMSQEYMAHGGQGLFTQAGFIDSSQDDGQQNPYGVNNPNLQSQGLPNSLYSQPFAHYNT
TvUPF1          ............................................................
DdUPF1   1238   NNNNNNNNNNNNNNNINNNNSKNQNQNQSPRRSPVVGTLQLHCKFDSQPNNNKHISDPNQ
GiUPF1          ............................................................
TgUPF1   1373   TPGAGKRGGPKSSDRDARNETRGLGESLGPANASGASDPDFVGRDSGCASRPNRAATVVS
PfUPF1   1499   SEQINSSKTQKGLKNTVLNKSSTSNTMVSQKDSIIVNDQKLNCDNLKKVSENTHIN....
```

```
TbUPF1        ..................................
ScUPF1        ..................................
HsUPF1        ..................................
AtUPF1  1229  QPLNLSGPQQSQPNQSSQNPKHPYNG........
TvUPF1        ..................................
DdUPF1  1298  PQISNPNMSIPLSQSLSFNDLSQEQFNKSNSRKD
GiUPF1        ..................................
TgUPF1  1433  EQLDDDSFTFIGSQVGF.................
PfUPF1        ..................................
```
