# Supplementary material for: Is There a Classical Nonsense-Mediated Decay Pathway in Trypanosomes?
Source: PLoS One. 2011 Sep 21;6(9):e25112. doi: 10.1371/journal.pone.0025112 (PMC3177853; doi:10.1371/journal.pone.0025112)
Supplement: Figure S2 — The strategy for replacement of the GPI-PLC gene with a mutant copy. In wild-type cells the GPI-PLC gene (pink) is flanked by HSP100 (green) and ß′-COP genes. The poly(A) site is marked as “a” and the two alternative spliced learder addition sites as black dots (“SL” for “spliced leader”). The panel below shows the loci in the double knockout line, and below that the construct that was used to return the full-length or PTC versions of GPI-PLC. The bottom panel shows the loci in cells containing the integrated return construct. (PDF) [file pone.0025112.s002.pdf]

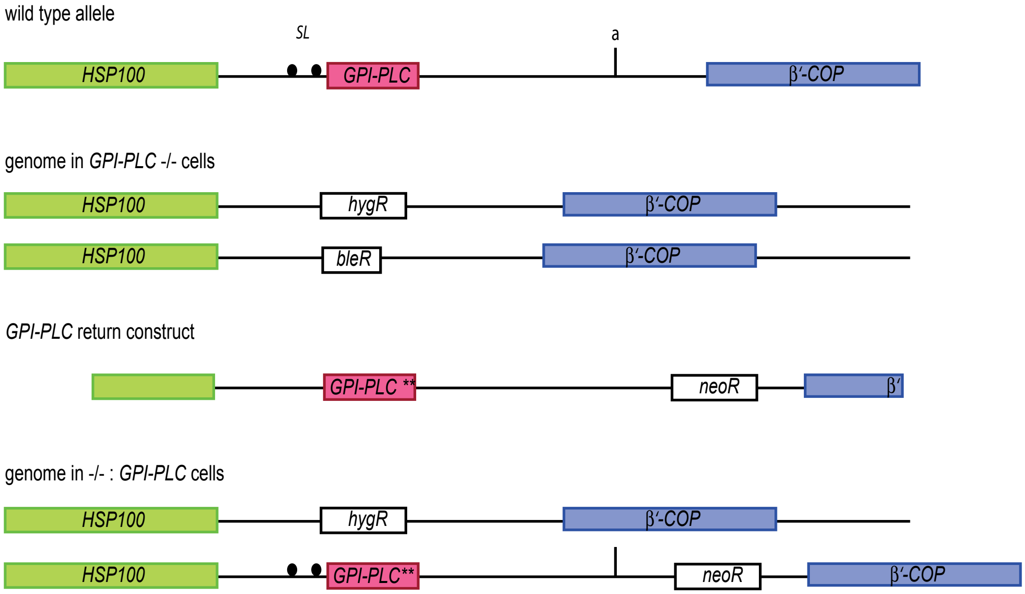

Supplementary Figure S2
The strategy for replacement of the *GPI-PLC* gene with a mutant copy. In wild-type cells the *GPI-PLC* gene (pink) is flanked by *HSP100* (green) and *ß'-COP* genes. The poly(A) site is marked as "a" and the two alternative spliced learder addition sites as black dots ("*SL*" for "spliced leader"). The panel below shows the loci in the double knockout line, and below that the construct that was used to return the full-length or PTC versions of *GPI-PLC*. The bottom panel shows the loci in cells containing the intergrated return construct.
