# Supplementary material for: Is There a Classical Nonsense-Mediated Decay Pathway in Trypanosomes?
Source: PLoS One. 2011 Sep 21;6(9):e25112. doi: 10.1371/journal.pone.0025112 (PMC3177853; doi:10.1371/journal.pone.0025112)
Supplement: Figure S3 — RNA degradation kinetics for selected CAT constructs, including the effects of UPF1 RNAi, UPF2 RNAi, or tethering of PABP. Results are shown as arithmetic mean ± standard deviation for 3 or more experiments, with all values at +2 h set as 100%. When two experiments were done, each is shown individually. The curves were fitted for time points from +2 h onwards. The half-lives shown were calculated separately for each individual experiment and – if there were three experiments - are presented as arithmetic mean ± standard deviation. The constructs used are indicated above the graphs; the presence of a tethered protein or induction of RNAi are also indicated. (PDF) [file pone.0025112.s003.pdf]

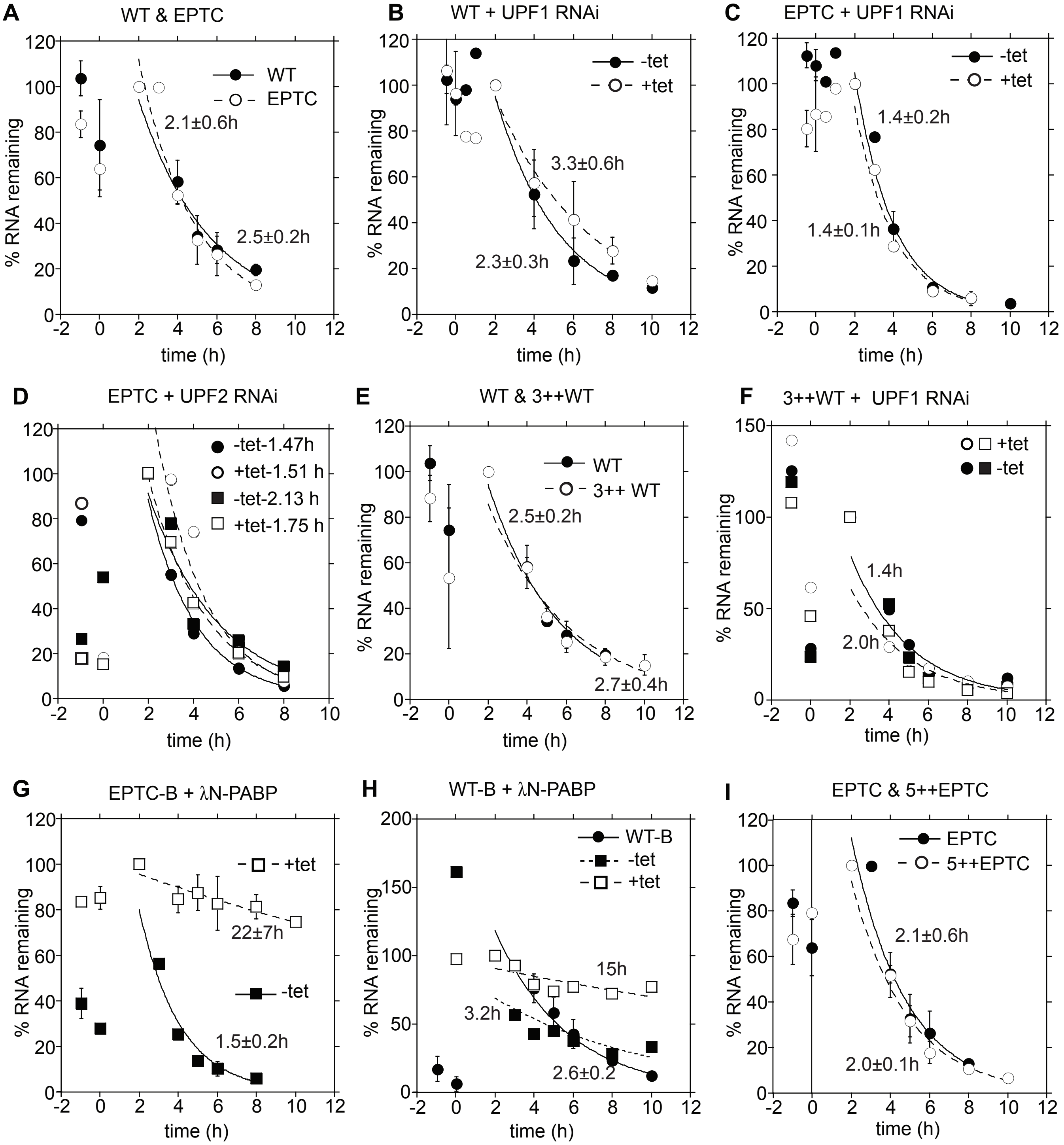

Supplementary Figure S3

RNA degradation kinetics for selected *CAT* constructs, including the effects of *UPF1* RNAi, *UPF2* RNAi, or tethering of PABP. Results are shown as arithmetic mean ± standard deviation for 3 or more experiments, with all values at +2h set as 100%. When two experiments were done, each is shown individually. The curves were fitted for time points from +2h onwards. The half-lives shown were calculated separately for each individual experiment and – if there were three experiments - are presented as arithmetic mean ± standard deviation. The constructs used are indicated above the graphs; the presence of a tethered protein or induction of RNAi are also indicated.
