# Supplementary material for: Is There a Classical Nonsense-Mediated Decay Pathway in Trypanosomes?
Source: PLoS One. 2011 Sep 21;6(9):e25112. doi: 10.1371/journal.pone.0025112 (PMC3177853; doi:10.1371/journal.pone.0025112)
Supplement: Figure S4 — Tethering of UPF1 to T7-transcribed CAT-B-ACT mRNA increases the mRNA degradation rate. The Figure show the results for two inependent experiments, done using the clones shown in Figure 5C, and as described for Figure S4. Expression of lambdaN-UPF1-flag was induced with tetracycline. In the absence of lambdaN-UPF1-FLAG induction, the half-life of the CAT-B-ACT mRNA was 15–20 min. This was surprisingly short. We do not understand why, but one possible explanation is that the mRNAs were made by T7 polymerase. Instability of a T7-produced CAT reporter with a different 3′-UTR has been previously observed; the reasons are unknown (Colasante et al., Mol Biochem Parasitol 151, 193–204.) Upon induction of lambdaN-UPF1-FLAG expression the half-life decreased further. (PDF) [file pone.0025112.s004.pdf]

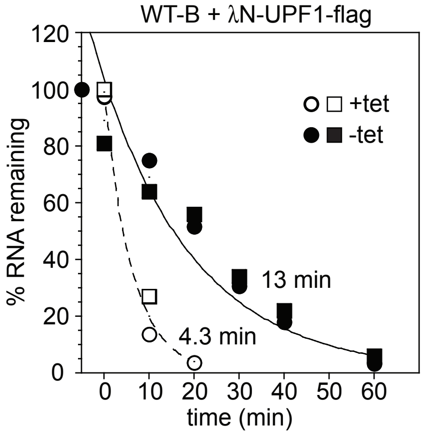

Supplementary Figure S4.
Tethering of UPF1 to T7-transcribed *CAT-B-ACT* mRNA increases the mRNA degradation rate.
The Figure show the results for two inependent experiments, done using the clones shown in Figure 5C, and as described for Figure S4. Expression of lambdaN-UPF1-flag was induced with tetracycline. In the absence of lambdaN-UPF1-FLAG induction, the half-life of the *CAT-B-ACT* mRNA was 15-20 min. This was surprisingly short. We do not understand why, but one possible explanation is that the mRNAs were made by T7 polymerase. Instability of a T7-produced *CAT* reporter with a different 3'-UTR has been previously observed; the reasons are unknown (Colasante et al., Mol Biochem Parasitol 151, 193-204.) Upon induction of lambdaN-UPF1-FLAG expression the half-life decreased further.
