# Supplementary material for: Is There a Classical Nonsense-Mediated Decay Pathway in Trypanosomes?
Source: PLoS One. 2011 Sep 21;6(9):e25112. doi: 10.1371/journal.pone.0025112 (PMC3177853; doi:10.1371/journal.pone.0025112)
Supplement: Figure S5 — RNAi targeting TbUPF1 has minor effects on the abundances of mRNAs from two loci. (A) shows a map of the region around Tb927.10.12900. The direction of transcription and the positions of various probes (a–d, dotted lines) used to identify the various RNAs is indicated. Estimated sizes and location of monocistronic RNAs, based on our own mapping and on RNASeq tag abundances shown at tritrypDB, are also shown. (B) Shows a Northern blot of RNA with and without RNAi targeting either XRNA or UPF1, using probe (a) (made using CZ3585, CZ3586). A Histone H4 probe (HISH4) serves as a control. Probe ‘c’ (made using oligonucleotides CZ3695, CZ3696) detected a single RNA migrating at 3.7 kb and probe ‘d’ (made using CZ3707, CZ3708) detected two RNAs of similar abundance, migrating at 2.6 kb and 1.8 kb. The band of 4.5 kb was detected only by probes (a) and (b). There is a tag gap in the middle of Tb10.389.0620, but no mapped spliced leader addition site. (C) The procyclin locus containing EP1 and EP2, with predicted transcripts based on mapped splicing sites. Alternative splicing of the procyclin EP2 RNA precursor is known to result in two transcripts. The dominant one, at 0.9 kb, has a short 5′-UTR preceding the EP2 initiation codon. The minor one, of 1.4 kb, has various very short ORFs upstream of the EP2 ORF while the 0.4 kb RNA contains these short ORFs alone: either of these might be a substrate for NMD. The location of the EP2 upstream probe used in (D) is indicated as a dotted line. (D) RNA was prepared from three independent bloodstream-form trypanosome clones (AnTat1.1. strain) with TbUPF1 hpRNAi, incubated with 100 ng/ml tetracycline for 2 days. The blot was probed with the EP2 upstream region indicated in (A). We detected the 0.9 kb EP mRNAs and three additional bands at 0.4, 1.4 and 2.3 kb. The 2.3 kb signal comigrates with rRNA so could either be a cross-hybridisation or a dicistronic precursor. The bands were all of approximately equal intensity: the mature EP mRN [file pone.0025112.s005.pdf]

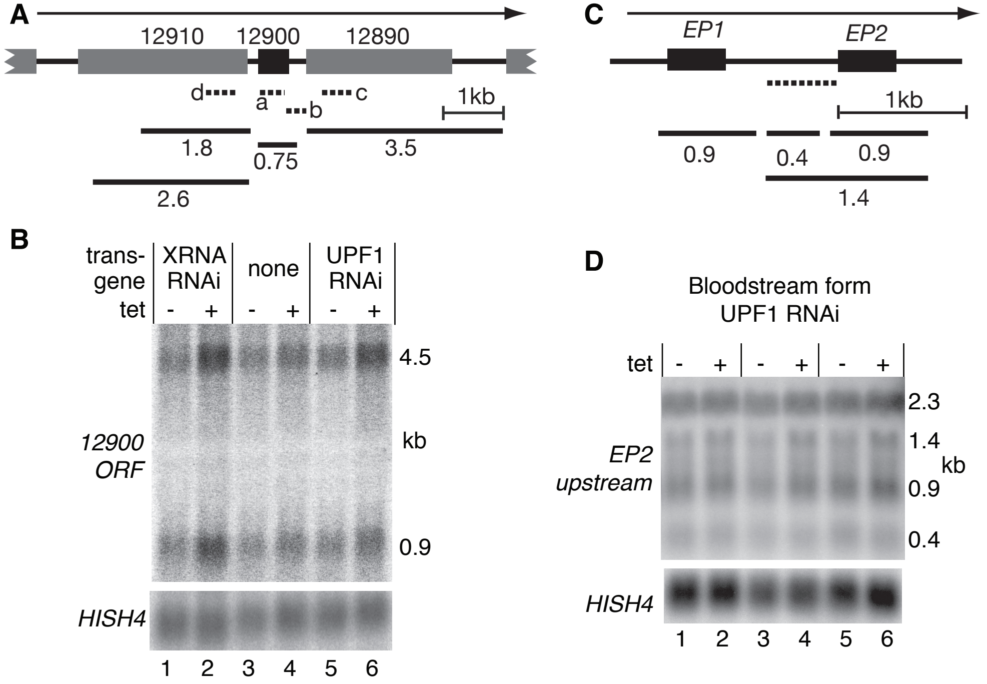

Supplementary Figure S5

RNAi targeting *TbUPF1* has minor effects on the abundances of mRNAs from two loci.

(A) shows a map of the region around Tb927.10.12900. The direction of transcription and the positions of various probes (a-d, dotted lines) used to identify the various RNAs is indicated. Estimated sizes and location of monocistronic RNAs, based on our own mapping and on RNASeq tag abundances shown at tritrypDB, are also shown.
(B) Shows a Northern blot of RNA with and without RNAi targeting either *XRNA* or *UPF1*, using probe (a) (made using CZ3585, CZ3586). A Histone H4 probe (*HISH4*) serves as a control. Probe 'c' (made using oligo-nucleotides CZ3695, CZ3696) detected a single RNA migrating at 3.7 kb and probe 'd' (made using CZ3707, CZ3708) detected two RNAs of similar abundance, migrating at 2.6kb and 1.8 kb. The band of 4.5 kb was deteceted only by porbes (a) and (b). There is a tag gap in the middle of Tb10.389.0620, but no mapped spliced leader addition site.
(C) The procyclin locus containing *EP1* and *EP2*, with predicted transcripts based on mapped splicing sites. Alternative splicing of the procyclin *EP2* RNA precursor is known to result in two transcripts. The dominant one, at 0.9kb, has a short 5'-UTR preceding the *EP2* initiation codon. The minor one, of 1.4 kb, has various very short ORFs upstream of the *EP2* ORF while the 0.4kb RNA contains these short ORFs alone: either of these might be a substrate for NMD. The location of the *EP2* upstream probe used in (D) is indicated as a dotted line.
(D) RNA was prepared from three independent bloodstream-form trypanosome clones (AnTat1.1. strain) with *TbUPF1* hpRNAi, incubated with 100ng/ml tetracycline for 2 days. The blot was probed with the *EP2* upstream region indicated in (A). We detected the 0.9 kb *EP* mRNAs and three additional bands at 0.4, 1.4 and 2.3 kb. The 2.3 kb signal comigrates with rRNA so could either be a cross-hybridisation or a dicistronic precursor. The bands were all of approximately equal intensity: the mature *EP* mRNA is very unstable in bloodstream forms. The level of the 1.4 kb *EP* mRNA increased 1.3-1.4 fold after *TbUPF1* depletion, whereas the levels of the monocistronic 0.9 kb mRNA and the 0.4kb RNA were unaffected.
The uORF-EP2 RNA probe was made by in vitro transcription using ^{32}P UTP and T7 RNA polymerase, from a PCR template carrying the T7 polymerase promoter sequence. (The primers were CZ3391 and CZ3392.)
