# Supplementary material for: Is There a Classical Nonsense-Mediated Decay Pathway in Trypanosomes?
Source: PLoS One. 2011 Sep 21;6(9):e25112. doi: 10.1371/journal.pone.0025112 (PMC3177853; doi:10.1371/journal.pone.0025112)
Supplement: Table S1 — Plasmids used in this study. (DOC) [file pone.0025112.s006.doc]

## SUPPLEMENTARY TABLE S1

Plasmids used in this study

| **CAT REPORTERS** | | |
| --- | --- | --- |
| pHD numbers | Name of construct | Description |
| 1991 | *WT-CAT* | The Neomycin phosphotransferase gene (*NPT*, N-terminally fused with a segment of the trypanosome beta-tubulin gene) was amplified with the primers CZ 3599 and CZ 3598 and cloned in the SpeI and StuI sites of pHD 1034, replacing the puromycin resistance gene. |
| 1992 | *EPTC-CAT*  (EPTC243) | PBS-SK with the *CAT* ORF was digested with EcoR1 and the ends were flushed using DNA pol I and re-ligated. The clones were sequenced. Insertion of 4nt at position 218 resulted in a frame shift, giving a nonsense codon ‘TGA’ at position 243 named early PTC. The entire *CAT* segment was subcloned in pHD1991. |
| 1993 | *LPTC-CAT*  (LPTC591) | PBS-SK with the *CAT* ORF was digested with NcoI and the ends were filled in using DNA pol I and re-ligated. The clones were sequenced. Insertion at position 519 gave a nonsense codon ‘TGA’ at position 591, named late PTC. The *CAT* segment subcloned in pHD 1991. |
| 2009 | EPTC 249 | Primers CZ 3611 and CZ 3610 were used to amplify a 189 bp ATG-less *GFP* fragment and cloned in EcoRI site of pHD 1991, introducing a stop codon at the start of *GFP* sequence. |
| 2010 | EPTC 393 | same as pHD 2009 but primers CZ 3609 and CZ 3610 were used and cloned in EcoRI site of pHD 1991, introducing a stop codon at the end of *GFP* sequence. |
|  | EPTC963 | MutGFP from pHD 2111 was amplified using primers CZ 3709 and CZ 3710 and cloned in EcoRI site of pHD 1991. |
| 2002 | *hp-WT-CAT* | Hairpin for translation block in 5’UTR of *CAT* gene: self- complementary oligonucleotide CZ 3595 (HP7) annealed, phosphorylated and cloned in HindIII of pHD1991. |
| 2003 | *hp-EPTC-CAT* | same as pHD 2002 but cloned in HindIII of pHD1992. |
| 2004 | *hp-LPTC-CAT* | same as pHD 2002 but cloned in HindIII of pHD1993. |
| 2081a | WTCAT with stop codon. | *CAT* ORF amplified using primers CZ 3590 and CZ 3678 and cloned in HindIII- BamHI sites of pHD1991. The 90 bp after the stop codon was deleted. The *CAT* ORF ends with a stop codon followed by BamHI. |
| 2081b | WTCAT without stop codon. | same as pHD 2081a but the *CAT* ORF was amplified using primers CZ 3590 and CZ 3677. The *CAT* ORF does not end with a stop codon. |
| 2083 | *CAT-GFP* ORF | Primers CZ 3679 and CZ 3676 were used to amplify the entire 720 bp *GFP* sequence with a start ATG and cloned in frame with *CAT* in the BamHI site of pHD 2081b. This code’s for a *CAT-GFP* fusion protein. |
| 2156 |  | pHD1437 + *NPT* as for 1991(1437 integrates into the tubulin locus, has a T7 promoter, a *CAT* ORF and a *HYG* resistance gene.) |
| 2164 | *WT-CAT* (pol II) | pHD2156 without T7 promoter, for pol II transcription of WT *CAT*. |
| 2223 | *EPTC-CAT*  (pol II) | CAT-EPTC from pHD1992 cloned into pHD2164, for pol II transcription of *EPTC-CAT*. |
| **UTRs EXTENSION CONSTRUCTS.** | | |
| 2006 | 5’+WT | Primers CZ 3613 and CZ 3612 were used to amplify an ATG-less 189 bp product of *GFP* and cloned in HindIII site of pHD 1991. |
| 2007 | 5’+EPTC | same as pHD 2006, the product cloned in HindIII site of pHD 1992. |
| 2008 | 5’+LPTC | same as pHD 2006, the product cloned in HindIII site of pHD 1993. |
| 2011 | 3’+WT | Primers CZ 3608 and CZ 3607 were used to amplify an ATG-less 189 bp product of *GFP* and cloned in BamHI site (3’ UTR) of pHD 1991. |
| 2012 | 3’+EPTC | Same as pHD 2011 but the product was cloned in BamHI site (3’ UTR) of pHD 1992. |
| 2013 | 3’+LPTC | Same as pHD 2011 but the product was cloned in BamHI site (3’ UTR) of pHD 1993. |
| 2084 | 3’++WTs | Primers CZ 3675 and CZ 3676 were used to amplify the entire 720 bp *GFP* sequence without a start ATG and cloned in the BamHI site of pHD 2081a. |
| 2111 | MutGFP | *GFP* ORF without ATG cloned in p*GEMT*. The 6 internal ATGs were mutated by overlap extension polymerase chain reaction (OE-PCR). For every ATG that was mutated a set of partial complementary oligonucleotides were designed which carried the desired substitution and were used to amplify two overlapping segments - upstream and downstream encompassing the mutation. The two overlapping PCR products for each mutation were purified and mixed; PCR amplified for few cycles without the primers and then further amplified using forward primer of the upstream region and reverse primer of the downstream region. The oligonucleotides were ATG-1 (CZ3661, CZ3662): ATG-2 (CZ 3663, CZ 3664); ATG-3 (CZ 3658, CZ 3657); ATG-4 (CZ 3659, CZ 3660); ATG-5 (CZ 3655, CZ 3656) and ATG-6 (CZ 3654). All are listed in Supplementary Table 1. The entire *GFP* gene was reconstructed by overlap of shorter fragments to bigger fragments by overlap extension and PCR amplification. The final 720 bp mutated *GFP* product was cloned in p*GEMT* and sequenced |
| 2112 | 5’++WT | The MutGFP was PCR amplified using primers CZ 3665 and CZ 3654 and cloned in HindIII site of pHD 1991. |
| 2113 | 5’++EPTC | same as pHD 2112 but cloned in HindIII site of pHD 1992. |
| 2114 | 5’++LPTC | same as pHD 2112 but cloned in HindIII site of pHD 1993. |
| 2115 | 3’++WTns | The MutGFP was PCR amplified using primers CZ 3675 and CZ 3676 and cloned in BamHI site of pHD 2081a. |
| 2116 | 3’++EPTC | The MutGFP was PCR amplified using primers CZ 3675 and CZ 3676 and cloned in BamHI site of pHD 1992. |
| 2117 | 3’++LPTC | same as pHD 2116 but the product was cloned in BamHI site of pHD 1993. |
| 2118 | 5’+++WT | The MutGFP was PCR amplified using primers CZ 3665 and CZ 3654 and inserted twice in HindIII site of pHD 1991. |
| 2119 | 5’+++EPTC | same as pHD 2118 but inserted twice in HindIII site of pHD 1992. |
| 2120 | 5’+++LPTC | same as pHD 2118 but inserted twice in HindIII site of pHD 1992. |
| **TARGET mRNAs FOR TETHERING ASSAYS.** | | |
| 1519 | 6 x BoxB | PBS-SK containing 6 B-Boxes (+3´UTRGlobin) (cloned with EcoRI - XhoI) |
| 2082 | *EPTC-BoxB* | 6x BoxB were cloned in EcoR1 site of 1991 with a stop at the beginning of the sequence. |
| 2084 | 3’++WTns-BoxB | 6x BoxB were cloned in BamH1 site of 3’++WTns (pHD 2115) after the GFPns. |
| 1520 | *CAT-BoxB* | pHD 1437 with B-Boxes from 1519 (cloned with BamH1 x XhoI) |
| 1522 | T7 promoter -*CAT-B-ACT* | pHD 1520 with *PAC* and *ACT* 3´UTR from pHD1034 (cloned with SpeI & SalI) |
| 1522hp | *hp-CAT-B-ACT* | Hairpin in 5’UTR of *CAT* gene: self-complementary oligonucleotide CZ 3595 (HP7) annealed, phosphorylated and cloned in HindIII of pHD 1522. |
| 1706 | T7 promoter -*CAT-MS2* | pHD1437 (BamHI*/*Sma I) + MS2 loop (BamHI/EcoRV) |
| 1937 | T7 promoter -*CAT-MS2-ACT* | *CAT-MS2* PCR product amplified from 1706 and replaced in pHD 1522 (HindIII/Xho1).Primers used CZ 3590 and CZ 3589. |
| **RNA INTERFERENCE CONSTRUCTS** | | |
| 1894 | *UPF1* hairpin RNAi | A 595 bp fragment of *UPF1* was amplified using primers CZ 3397  5’-GAGAAGATCTCTCGAGGGTACCAGCGGTTCTCACAT-3' and CZ 3398 5’-CGGATATCGTCGACTTGATACGTTATCACCGCGA-3'. The fragment was cloned in XhoI-SalI of pHD 1144, then in inverse orientation in BglII-EcoRV. The hairpin was released by partial digestion with XhoI, end filled with klenow enzyme, and then digested with BglII. The insert released was cloned in HpaI-BamHI sites of pHD 1146. |
| P2T7 *UPF1* RNAi | *UPF1* p2T7 RNAi | The entire *UPF1* open reading frame was cloned into p2T7-177. |
| 1872 | *UPF2* hairpin RNAi | An approximate 700 bp PCR fragment of *UPF2* was amplified using primers CZ3395  5'-GAGAAGATCTGCATGCTGAGCTCAAGCGTCCTCCAGTTCC-3' and CZ 3396  5'-CGGAATTCGTCGACCAGCTGCGGTAGTAGAAGGG-3'  The fragment was cloned in SphI-SalI of pHD 1144, then again in inverse orientation in BglII-EcoRI sites. The hairpin insert was excised by digesting with HindIII-BglII and then cloned into pHD 1146 in HindIII-BamHI. |
| **UPF1 EXPRESSION CONSTRUCTS** | | |
| 1743 | *λN-GFP-TAP* | pHD918+ lambdaN peptide (HindIII/ApaI) + *EGFP* (ApaI/HpaI) |
| 1807 | *λN-UPF1-FLAG* | Two self complementary oligonucleotides coding for the flag tag were annealed and cloned in the HpaI site of *UPF1* in p*GEMT* vector and sequenced.  CZ3557 5'-GATATCGACTACAAGGACGATGACGACAAAGTT-3’  CZ3556 5'-AACTTTGTCGTCATCGTCCTTGTAGTCGATATC-3' .  The entire ORF of *UPF1-FLAG* was amplified using CZ3567 and CZ3568 5'-CTTAGGATCCCTAAACTTTGTCGTCATCGTCC-3'  and cloned in ApaI and BamHI sites of pHD1743. |
| 1808 | *UPF1-FLAG* | The *UPF1-FLAG* gene was PCR amplified from pHD 1807 and subcloned in pHD 678 at the ApaI and BamHI sites.  CZ3567 5'-GCTAGGGCCCAGAATGTTCAGTGAGCATGCTAG-3'  CZ3568 5'-CTTAGGATCCCTAAACTTTGTCGTCATCGTCC-3' |
| 1809 | *UPF1-FLAG*(R→C) mutation | The downstream region from nt 2231 of the *UPF1* ORF to the end of the *FLAG* tag was amplified using pHD1808 and primers (underlined TGC is the mutation and the *FLAG* primer includes a BamH1 site):  CZ3562 5'-CGTGCGTTTGCAGCAATCACCGTCAG-3'  CZ3568 5'-CTTAGGATCCCTAAACTTTGTCGTCATCGTCC-3'  In addition an upstream region from nucleotide position 797 to the mutant segment, ending at position 2250, was amplified using primers:  CZ 3558 5'-GCAGTCTATCTGGCACTATCACTG-3  CZ3563 5'-GTGATTGCTGCAAACGCACGACAAAATTATG-3'  The PCR products were purified and mixed in equal proportion. The first 5 cycles of PCR amplification were carried out without any primers followed by the addition of primer set CZ 3558 and CZ 3568 and further amplified for 25 cycles.  The XhoI-BamHI fragment with the mutation was then replaced into the wild-type fragment of *UPF1* gene in pHD 1808. |
| 2079 | *UPF2-myc* | The ORF of *UPF*2 was amplified from genomic DNA of *T.brucei* 449 cells using primers CZ 3617 and CZ 3616 and cloned in HindIII and HpaI sites of pHD 1700. |
| 2080 | *In situ* V5 tag for *UPF1.* | A portion of the 5’ UTR using primers CZ 3673 and CZ 3674 and the coding region of *UPF1* using primers CZ 3671 and CZ 3672 was amplified and cloned in Bla-V5 vector. The region from 5’UTR to the CDS of *UPF2* including the blasticidin resistance marker was cut out and transfected in 1313 procyclic cells. |
| **PABP and GFP expression constructs** | | |
| 2198 | *PABP1myc* | *PABP1* (Tb09.211.0930) was PCR amplified using CZ 3652 and CZ 3653 from genomic DNA and cloned in Apa1/BamH1 of pHD 1700. |
| 2199 | *λNPABP1myc* | *PABP1myc* was PCR amplified using CZ 3652 and CZ 3638 from pHD 2198 and cloned in Apa1/BamH1 of pHD 1743. |
| 2200 | *λNPABP2myc* | *PABP2* (Tb09.211.2150) was PCR amplified using CZ 3650 and CZ 3651 from genomic DNA and cloned in Hpa1/BamH1 of pHD 2199. |
| 2201 | *λN-myc* | Two oligonucleotides CZ 3928 and CZ 3929 were annealed, kinased and cloned in Hpa1/BamH1 of pHD 2199 replacing *PABP1*. |
| 2202 | *λNGFPmyc* | *GFP* was amplified using CZ 3930 and CZ 3931 and cloned in Hpa1 of pHD 2201 |
