# Supplementary material for: Is There a Classical Nonsense-Mediated Decay Pathway in Trypanosomes?
Source: PLoS One. 2011 Sep 21;6(9):e25112. doi: 10.1371/journal.pone.0025112 (PMC3177853; doi:10.1371/journal.pone.0025112)
Supplement: Table S2 — Oligonucleotides used in this study. (DOC) [file pone.0025112.s007.doc]

***SUPPLEMENTARY TABLE S2***

Oligonucleotides used in this study.

| **oligo name** | **CZ nos** | | **sequence** |
| --- | --- | --- | --- |
| **hpRNAi of UPF1 & UPF2** | | | |
| SLupf2for | 3395 | | 5'-GAGAAGATCTGCATGCTGAGCTCAAGCGTCCTCCAGTTCC-3' |
| SLupf2rev | 3396 | | 5'-CGGAATTCGTCGACCAGCTGCGGTAGTAGAAGGG-3' |
| NM1for | 3397 | | 5'-GAGAAGATCTCTCGAGGGTACCAGCGGTTCTCACAT-3' |
| Nm1rev | 3398 | | 5'-CGGATATCGTCGACTTGATACGTTATCACCGCGA-3' |
| **Expression constructs of UPF1, upf1-1, UPF2, PABP1, PABP2, GFP.** | | | |
| fla-rev | 3556 | | 5'-AACTTTGTCGTCATCGTCCTTGTAGTCGATATC-3' |
| fla-for | 3557 | | 5'-GATATCGACTACAAGGACGATGACGACAAAGTT-3' |
| UPF-INT_FOR | 3558 | | 5'-GCAGTCTATCTGGCACTATCACTG-3' |
| mutupf-2_for | 3562 | | 5'-CGTGCGTTTGCAGCAATCACCGTCAG-3' |
| mutupf-1_rev | 3563 | | 5'-GTGATTGCTGCAAACGCACGACAAAATTATG-3' |
| NMD1_REV | 3565 | | 5'-CTCGAGGTTAACAGACTCACCAGGCAAGAACAATGG-3' |
| Nmd1_for | 3567 | | 5'-GCTAGGGCCCAGAATGTTCAGTGAGCATGCTAG-3' |
| Flag_rev | 3568 | | 5'-CTTAGGATCCCTAAACTTTGTCGTCATCGTCC-3' |
| Upf2for | 3616 | | 5'-CATAAGCTTATGAGCTCAAGCGTCCTC-3' |
| Upf2rev | 3617 | | 5'-AATTCAGTTAACGCGACGCTCAGATTCCTG-3' |
| Apa1U1cdsrev | 3671 | | 5’-TAGGGCCCTCAGTGATAGTGCCAGATAG-3’ |
| Xho1U1cdsfor | 3672 | | 5’-TTCTCGAGATGTTCAGTGAGCATGCTAG-3’ |
| Sac11U1utrfor | 3673 | | 5’-TTTTTCCGCGGTGATTATTTCGCCTTATCAGG-3’ |
| Xba1U1utrrev | 3674 | | 5’-AGCTTCTAGAAACAACTTTTCAGAAACACGG-3’ |
| PAB2150for | 3650 | | 5'-TTTCCCGTTAACATGGCTGCATTTGCTGCTG-3' |
| PAB2150rev | 3651 | | 5'-CACGGATCCCATGCCAATGTGACGGTTG-3' |
| PAB0930for | 3652 | | 5'-GAGGGCCCGTTAACATGACAATCGCTGCACAGG-3' |
| PAB0930rev | 3653 | | 5'-CCAGGATCCAGCGCTTGAGGCGTGTAC-3' |
| FORP-myc | 3928 | | 5’-AACGGTGCTGCCTCCG-3’ |
| REVP-myc | 3929 | | 5’-GGATCCGGAGGCAGCACCGTT-3’ |
| GFPfor | 3930 | | 5'-AACGAGTTAACATGGACGCACAAACACGACG-3' |
| GFPrev | 3931 | | 5'-ACTAAGTTAACTTAGTGATGGTGATGGTGATG-3' |
| **CAT constructs** | | | |
| CAT-for | 3590 | | 5'-ACATTAAGCTTCGACGAGATTTTCAGGAG-3' |
| CATrev | 3677 | | 5'-ATGGATCCCGCCCCGCCCTGCC-3' |
| CATrev2 | 3678 | | 5'-ATGGATCCTTACGCCCCGCCCTGC-3' |
| MS2-rev | 3589 | | 5'-TAATCTCGAGCTCCTGCTACGCCGCCTC-3' |
| HP7 | 3595 | | 5'-AGCTTGGGGCGCGTGGTGGCGGCTGCAGCCGCCACCACG  CGCCCCA-3' |
| Neorev | 3598 | | 5'-CATATAGGCCTTCAGAAGAACTCGTCAAGAAGG-3' |
| Neofor | 3599 | | 5'-ACACTAGTAACGAATATGGTGGAACAAGATGGATTGC  ACGCAGGTTCTC-3' |
| BamHI-HpaIrev | 3607 | | 5'-CATGGATCCGTTAACTGTCGGCGATGATATAGAC-3' |
| BamHI-XbaIfor | 3608 | | 5'-CATGGATCCTCTAGATTCAAGGACGACGGCAAC-3' |
| EcoRI-XhoIfor | 3609 | | 5'-CATGAATTCCTCGAGTTCAAGGACGACGGCAAC-3' |
| EcoRI-BclIrev | 3610 | | 5'-CATGAATTCTGATCAGTCGGCGATGATATAGAC-3' |
| EcoRI-BclIfor | 3611 | | 5'-CATGAATTCTGATCATTCAAGGACGACGGCAAC-3' |
| HindII-XhoIrev | 3612 | | 5'-CATAAGCTTCTCGAGTGTCGGCGATGATATAGAC-3' |
| HindII-BclIfor | 3613 | | 5'-CATAAGCTTTGATCATTCAAGGACGACGGCAAC-3' |
| **GFP mutation & deletions** | | | |
| primer7rev | 3654 | | 5'-CTAAGCTTGTACAGCTCGTCAATGCC-3' |
| Primer6rev | 3655 | | 5'-CCAGCAGGACCTTGTGATC-3' |
| primer6for | 3656 | | 5'-GATCACAAGGTCCTGCTGG-3' |
| primer5rev | 3657 | | 5'-GTAGCCTTCGGGCAAGGCG-3' |
| Primer5for | 3658 | | 5'-CtTGCCCGAAGGCTAC-3' |
| primer4rev | 3659 | | 5'-GTCGGCCAAGATATAGACG-3' |
| primer4for | 3660 | | 5'-CGTCTATATCTTGGCCGAC-3' |
| primer3rev | 3661 | | 5'-GGTGGCAACGCCCTC-3' |
| primer3for | 3662 | | 5'-GGGCGTTGCCACCTAC-3' |
| primer2rev | 3663 | | 5'-CTGCTTGATGTGGTCGG-3' |
| primer2for | 3664 | | 5'-CCGACCACATCAAGCAG-3' |
| primer1for | 3665 | | 5'-CTAAGCTTGTGAGCAAGGGCGAG-3' |
| GFPfor | 3675 | | 5'-ATTAGATCTTGGTGAGCAAGGGCG-3' |
| GFP rev | 3676 | | 5'-ATTGGATCCTTACTTGTACAGCTCGTCC-3' |
| GFP2FOR | 3679 | | 5'-ATTAGATCTATGGTGAGCAAGGGCG-3' |
| mGFPEcoRIF | 3709 | | 5’-CTTGAATTCGTGAGCAAGGGCGAG-3’ |
| mGFPEcoRIR | 3710 | | 5’-CTAGAATTCTTACTTGTACAGCTCGTC-3’ |
| **qPCR primers of CAT & NPT.** | | | |
| cat-rev | | 3633 | 5'-TTCATTAAGCATTCTGCCGACAT-3' |
| cat-for | | 3634 | 5'-GCCGCTGGCGATTCAG-3' |
| neo-rev | | 3635 | 5'-GCCGGATCAAGCGTATGC-3' |
| neo-for | | 3636 | 5'-CTCCTGCCGAGAAAGTATCCA-3' |
| **Probes** | | | |
| Tb10.389.0620for | | 3585 | 5'-ATGCTGCACTGTTCACAGTG-3' |
| Tb10.389.0620rev | | 3586 | 5'-TAATACGACTCACTATAGGGAGTAGTGTACCAATGA  GAACCTC-3 |
| 620for1 | | 3626 | 5'-CAAGAGTGCAAACATATCGC-3' |
| IGR630rev | | 3667 | 5'-TTCTAATACGACTCACTATAGGCGGATACATGTTGCCATC-3' |
| 610for | | 3707 | 5’-TTGCACCACGAATACTGCTC-3’ |
| 610rev | | 3708 | 5’-CGGAAGGAGGTATCCAACAA-3’ |
| HP0630for | | 3695 | 5'-CATAATGCGTCCTTGGTCCT-3' |
| HP0630rev | | 3696 | 5'-TTTGTCTGGGTCCTGGAAAC-3' |
| EP_FALSEUTR_F | | 3391 | 5'-CTTCTAAACCTTCAGGCCAG-3' |
| EP_FALSEUTR_R | | 3392 | 5'-GAATTCTAATACGACTCACTATAGGGAACGAGGTG  CCATTG-3' |
